# Aberrantly expressed miR-582-3p maintains lung cancer stem cell-like traits by activating Wnt/β-catenin signalling

**DOI:** 10.1038/ncomms9640

**Published:** 2015-10-15

**Authors:** Lishan Fang, Junchao Cai, Baixue Chen, Shanshan Wu, Rong Li, Xiaonan Xu, Yi Yang, Hongyu Guan, Xun Zhu, Le Zhang, Jie Yuan, Jueheng Wu, Mengfeng Li

**Affiliations:** 1 Department of Microbiology, Sun Yat-sen University, Guangzhou, Guangdong 510080, China; 2 Key Laboratory of Tropical Disease Control, Sun Yat-sen University, Ministry of Education, Guangzhou, Guangdong 510080, China; 3 Department of Pharmacology, Zhongshan School of Medicine, Sun Yat-sen University, Guangzhou, Guangdong 510080, China; 4 Department of Endocrinology and Diabetes Center, The First Affiliated Hospital of Sun Yat-sen University, Guangzhou, Guangdong 510080, China; 5 Key Laboratory of Functional Molecules from Oceanic Microorganisms, Sun Yat-sen University, Department of Education of Guangdong Province, Guangzhou, Guangdong 510080, China

## Abstract

Cancer stem cells (CSCs) are involved in tumorigenesis, tumour recurrence and therapy resistance and Wnt signalling is essential for the development of the biological traits of CSCs. In non-small cell lung carcinoma (NSCLC), unlike in colon cancer, mutations in β-catenin and APC genes are uncommon; thus, the mechanism underlying the constitutive activation of Wnt signalling in NSCLC remains unclear. Here we report that miR-582-3p expression correlates with the overall- and recurrence-free-survival of NSCLC patients, and miR-582-3p has an activating effect on Wnt/β-catenin signalling. miR-582-3p overexpression simultaneously targets multiple negative regulators of the Wnt/β-catenin pathway, namely, AXIN2, DKK3 and SFRP1. Consequently, miR-582-3p promotes CSC traits of NSCLC cells *in vitro* and tumorigenesis and tumour recurrence *in vivo*. Antagonizing miR-582-3p potently inhibits tumour initiation and progression in xenografted animal models. These findings suggest that miR-582-3p mediates the constitutive activation of Wnt/β-catenin signalling, likely serving as a potential therapeutic target for NSCLC.

## Abstract (web summary)

Despite the absence of frequent mutations of key components of Wnt/β-catenin signalling, this pathway is often constitutively activated in non-small cell lung carcinoma. In this study, the authors demonstrate the inhibitory effect of miR-582-3p on negative regulators of the Wnt/β-catenin pathway which is consequently triggered to sustain lung tumour growth.

## Graphical abstract

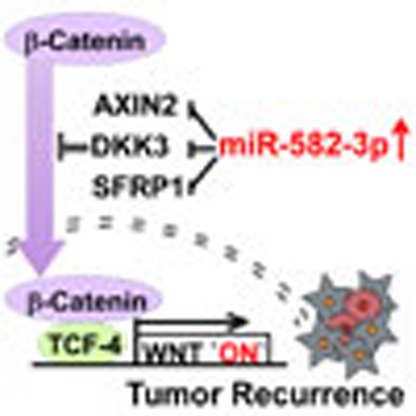

Lung cancer remains a leading cause of cancer-associated death worldwide. Non-small cell lung cancer (NSCLC), which includes adenocarcinoma, squamous cell carcinoma and large cell carcinoma, accounts for over 80% of all lung cancer cases^1^. Despite advances made in therapeutic technologies and strategies, the prognosis of this disease remains poor, with the 5-year survival rate of NSCLC (all stages combined) being only ∼15% (ref. 2) Tumour recurrence is common and represents a major obstacle to the improvement of patient survival. Even after *R*_0_ resection, the long-term survival rate for NSCLC is <50% (ref. 3). Thus, elucidation of the mechanisms underlying recurrence is fundamental for the development of new therapeutic treatments in NSCLC.

Cancer stem cells (CSCs) play important roles in tumour recurrence, metastasis, drug resistance and other malignant phenotypes of human cancer. The development of CSCs and maintenance of their ‘stemness' are associated with aberrations of several molecular cascades involving signalling triggered by Notch^4^, Hedgehog^5^ and Wnt^6^. Among these pathways, hyperactivation of Wnt-mediated signalling has been extensively identified as one of the most frequent events occurring in the CSCs of a variety of tumour types^7^,^8^. Activated Wnt/β-catenin signalling was reported to be essential for the self-renewal capacity and drug-resistant properties of human acute leukaemia stem cells, leading to poor patient survival^9^. Furthermore, a high level of Wnt activity in colon cancer cells stimulated by microenvironmental factors functionally designates the CSC population^10^. Conversely, ablation of the β-catenin gene results in loss of CSCs and complete tumour regression in cutaneous cancer^11^.

Intrinsic multilevel regulation of the Wnt signalling pathway has been well recognized and widely studied. Wnt signalling is triggered when active Wnt ligands bind to their membrane receptors frizzled (FZD) and LDL receptor-related protein 5 or 6 (LRP5/6), resulting in dissociation of β-catenin from the ‘destruction complex' consisting of Axin, APC, CK1α and GSK-3β. Consequently, stabilized cytosolic β-catenin translocates to the cell nucleus to form the β-catenin-LEF/TCF complex and thus induces the transcription of various downstream genes implicated in tumorigenesis. However, in the absence of Wnt ligand stimulation, cytosolic β-catenin is sequestrated in the ‘destruction complex'; subsequently, β-catenin is degraded and Wnt signalling is inactivated. Moreover, the initiation process of Wnt ligand binding to receptors can be directly blocked by extracellular antagonistic proteins^12^. Wnt antagonists can be divided into two groups, with one group containing the sFRP family members WIF-1 and Cerberus, which inhibit Wnt signalling by directly binding Wnt, and the other group including the Dickkopf (DKK) family, whose members suppress Wnt signalling by binding the LRP5/LRP6 components of the Wnt receptor complex^13^.

Notably, dysregulation of the abovementioned negative regulators and abnormal Wnt signalling have been found in NSCLC^14^. For example, silencing AXIN2, a key component of the ‘destruction complex', leads to nuclear β-catenin accumulation and lung tumorigenesis enhancement^15^. SFRP1, a member of the sFRP family and a Wnt antagonist, is significantly downregulated and has tumour-suppressive effects in lung cancer^16^. Another Wnt antagonist, DKK3, which is reported to be a tumour suppressor in a variety of malignancies, is able to induce lung cancer cell apoptosis^17^. In NSCLC, aberrant Wnt signalling correlates with tumour recurrence and poor patient survival^18^,^19^. However, unlike in colon cancer, mutations in β-catenin and APC genes appear to be uncommon in NSCLC according to previously reported studies^20^,^21^ and according to The Cancer Genome Atlas (TCGA) data sets, suggesting that epigenetic mechanisms might be involved in maintaining constitutive activation of Wnt/β-catenin signalling cascades in NSCLC cells.

MicroRNAs (miRNAs or miRs) are a class of small non-coding RNAs that mediate post-transcriptional repression of target genes through their interactions with 3′-untranslated regions (UTRs)^22^. Distinct miRNAs have been found to directly regulate the Wnt/β-catenin pathway in human malignancies including breast cancer^23^, liver cancer^24^ and colon cancer^25^. In NSCLC, members of the miR-29 family have been reported to reduce the expression of two DNA methyltransferases, namely, DNMT3A and DNMT3B, to abrogate WIF-1 promoter methylation, consequently upregulating WIF-1 expression and leading to Wnt signalling suppression. This finding suggests the importance of decreased expression of the miR-29 family in maintaining WIF-1 promoter methylation and Wnt signalling overactivation. However, whether dysregulation of miRNAs directly interferes with important Wnt signalling components to aberrantly activate this signalling pathway in NSCLC remains unclear. In the current study, we identify miR-582-3p as an important miRNA that is closely associated with NSCLC recurrence and poor patient survival. miR-582-3p is also functionally linked to the activation of the Wnt/β-catenin pathway and to the consequent increase in the CSC subpopulations in NSCLC both *in vitro* and *in vivo*. Our data suggest that miR-582-3p may play an important role in maintaining the stem cell-like properties of a subset of NSCLC cells.

## Results

### miR-582 correlates with NSCLC recurrence and patient survival

In an attempt to identify miRNAs associated with NSCLC recurrence and patient survival, we analysed the miRNA expression profiles of patients with adenocarcinoma and squamous cell carcinoma for whom recurrence-free survival (RFS) and overall survival (OS) data were available in the lung cancer data set of TCGA. Using censored survival analysis of the Significance Analysis of Microarrays (SAM) module in MeV4.9, 15 distinctly expressed miRNAs were found to be closely correlated with either RFS or OS, corresponding to a median expected false discovery rate (FDR) of 10% (Table 1), among which miR-99a and miR-196a-2 have been previously reported to be of clinical significance in NSCLC^26^,^27^. Interestingly, miR-582 emerged as the only miRNA that was significantly correlated with both RFS and OS.

**Figure 1:**
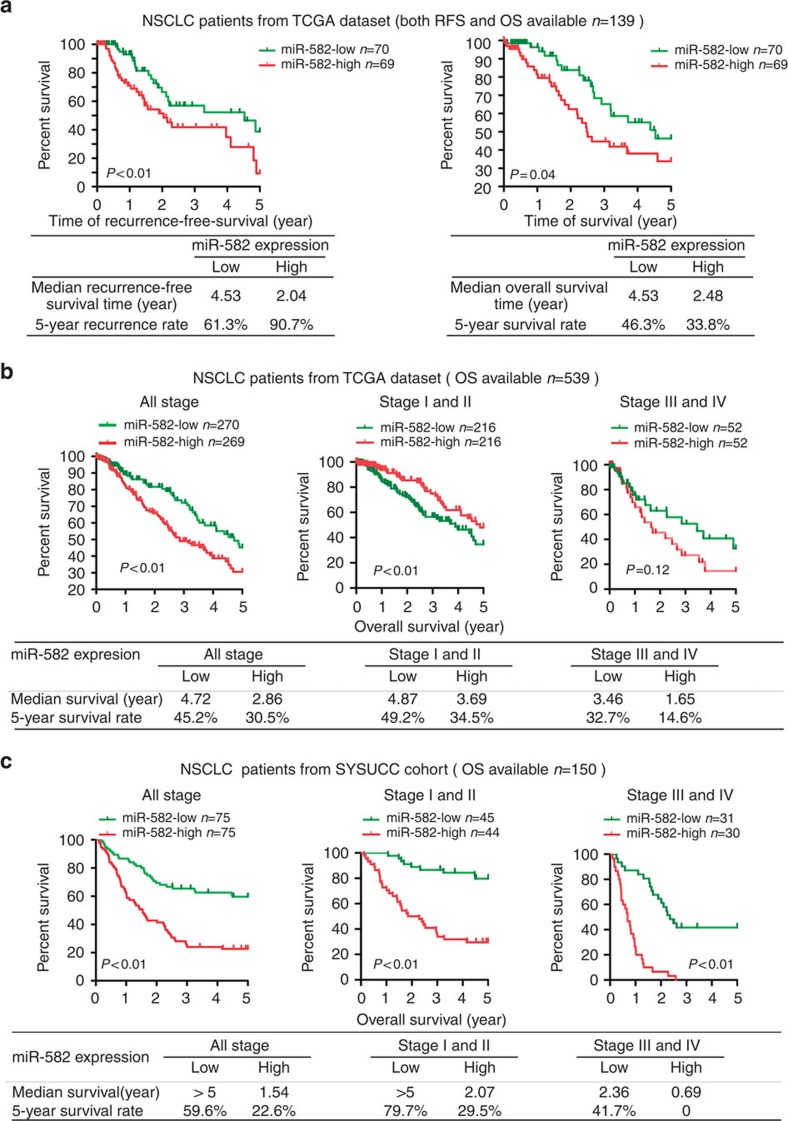
Upregulation of miR-582 in NSCLC is correlated with recurrence and poor prognosis. (**a**) Kaplan–Meier analysis of the 5-year RFS (left panel) and OS (right panel) of patients with NSCLC for whom both RFS and OS information was available in the TCGA lung cancer data set. The patients were stratified by high (greater than the median, *n*=69) versus low (greater than or equal to the median, *n*=70) expression of miR-582. (**b**,**c**) Kaplan–Meier analysis of the correlation between the miR-582 level and the 5-year OS of 539 patients with NSCLC in the TCGA cohort (**b**) and of 150 patients with NSCLC in the SYSUCC cohort (**c**), which is further analysed for 432 NSCLC patients in stages I–II and 104 patients in stages III–IV in the TCGA lung cancer cohort (**b**) and 89 patients at stages I–II and 61 patients at stages III–IV in the SYSUCC cohort (**c**).

More specifically, patients stratified by a median cutoff of miR-582 expression with higher miR-582 expression (>median) had shorter RFS and OS than those expressing lower levels of miR-582 (≤median; log-rank test, *P* <0.01 and *P*=0.04, respectively; Fig. 1a). Furthermore, in a set of 539 NSCLC patients in the TCGA data set (Supplementary Table 1) for whom OS data were available, the median survival times of patients with higher and lower miR-582 expression, respectively, were 2.86 and 4.72 years, and the cumulative 5-year survival rates for the high- and low-miR-582 cases were 30.5% and 45.2%, respectively (log-rank test, *P*<0.01, Fig. 1b). Similar results were also obtained in the Sun Yat-sen University Cancer Center (SYSUCC) cohort (log-rank test, *P*<0.01, Fig. 1c); clinical information for this cohort is summarized in Supplementary Table 2. Moreover, early-stage (stages I and II together) and late-stage (stages III and IV together) patients with high miR-582 expression had shorter survival durations than those with low miR-582 expression in both the TCGA lung cancer and the SYSUCC cohorts, suggesting that miR-582 level might be indicative of the prognosis of NSCLC patients at various clinical stages. Notably, in the TCGA lung cancer cohort, although the difference in OS for patients in late stages did not reach statistical significance (log-rank test, *P*=0.12), which may have been confounded by the low incidence of death and the small number of censored cases in these subgroups, patients with tumours exhibiting high and low miR-582 expression had median survival times of 1.65 and 3.46 years, respectively, and the cumulative 5-year survival rates for the high- and low-miR-582 subgroup patients were 14.6% and 32.7%, respectively (Fig. 1b). Furthermore, the univariate analysis showed that an increased expression of miR-582 was associated with an increased risk of death in both the TCGA lung cancer and SYSUCC cohorts (Cox regression, TCGA cohort, *P*=0.005; SYSUCC cohort, *P*<0.001). In the multivariate analysis, after adjusting for the patient's age at diagnosis, gender, and tumour stage, miR-582 expression was significantly associated with a shorter OS in patients with NSCLC independent of tumour pathological types and TNM staging (Cox regression, TCGA cohort: hazard ratio (HR), 1.85; 95% confidence interval (95% CI), 1.25-2.73; *P*=0.002 and SYSUCC cohort: HR, 2.26; 95% CI, 1.39–3.68; *P*=0.001; Supplementary Tables 3 and 4). These data warrant further investigation to demonstrate the prognostic value of miR-582 expression as a clinically useful independent indicator for outcome evaluation of NSCLC patients.

### miR-582-3p is overexpressed in NSCLC cell lines and tissues

**Figure 2:**
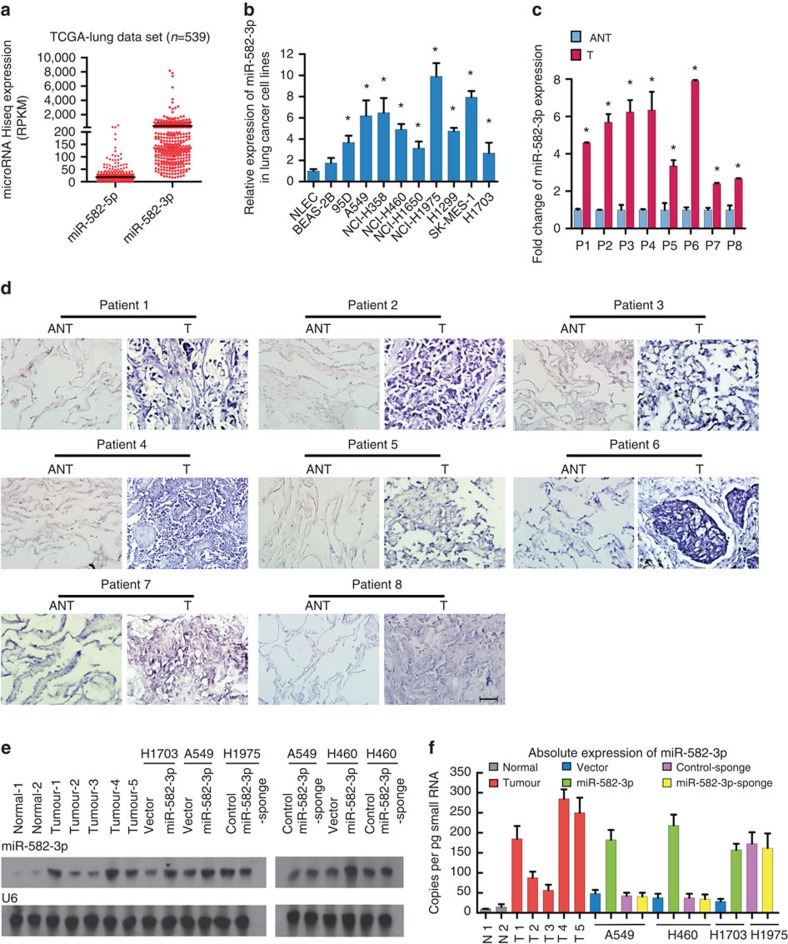
miR-582-3p is overexpressed in NSCLC cell lines and tissues. (**a**) Expression of miR-582-3p and miR-582-5p in human lung cancer clinical specimens of human lung cancer using the TCGA miRNA HiSeq expression array data. (**b**) RT–qPCR analysis of miR-582-3p expression in NLEC normal lung epithelial cells, including primary NLECs, the immortalized human bronchial epithelial cell line BEAS-2B, and a panel of eight human NSCLC cell lines. (**c**) Relative expression of miR-582-3p in 8 pairs of NSCLC tumour tissues (T) and their corresponding adjacent non-cancerous tissues (ANT). (**d**) miR-582-3p is upregulated in all 8 NSCLC tumor specimens compared with paired adjacent non-tumour tissue based on ISH. Scale bar, 50 μm. (**e**) Northern blotblotting analysis of miR-582-3p in the indicated tissues and cell lines. U6 was used as an internal reference. (**f**) Absolute quantification of miR-582-3p expression in the indicated tissue specimens and cell lines using real-time PCR and a standard curve.. Each bar represents the mean±s.e.m. derived from three independent experiments. A two-tailed Student's *t*-test was used for statistical analysis (*P<0.05).

Because the expression levels of miR-582-5p were much lower than the expression levels of miR-582-3p in the TCGA miRNA HiSeq expression array (Fig. 2a), we aimed to investigate the potential role of miR-582-3p in NSCLC progression. As shown in Fig. 2b,c, miR-582-3p was ubiquitously expressed at higher levels in a panel of 9 NSCLC cell lines and in 8 NSCLC tumor specimens, respectively, than in normal lung epithelial cells (NLECs) and in paired adjacent non-tumour tissue, which was further confirmed by *in situ* hybridization (ISH) analysis showing that miR-582-3p staining was only marginally detectable in normal lung tissues, in contrast to markedly overexpressed miR-582-3p in cytoplasmic areas of lung cancer lesions (Fig. 2d). In parallel, in the SYSUCC cohort, miR-582-3p levels remained relatively low in stage I and II tumours but were elevated in stage III tumours and further increased in stage IV tumours (Supplementary Fig. 1). Furthermore, the absolute miR-582-3p levels, as determined by northern blot analysis and absolute quantification via real-time (reverese transcription) RT–PCR, were significantly higher in NSCLC tissues/cell lines than in normal lung tissues and cells. More specifically, the absolute amount of miR-582-3p was approximately 50–300 copies per pg of small RNA in lung cancer tissues or cell lines compared with 9–15 copies per pg of small RNA (approximately one cell according to previous reports^28^) in normal lung tissues (Fig. 2e,f). Collectively, these data indicate that miR-582-3p is markedly upregulated in NSCLC.

### miR-582-3p enhances tumorigenicity and tumour recurrence

**Figure 3:**
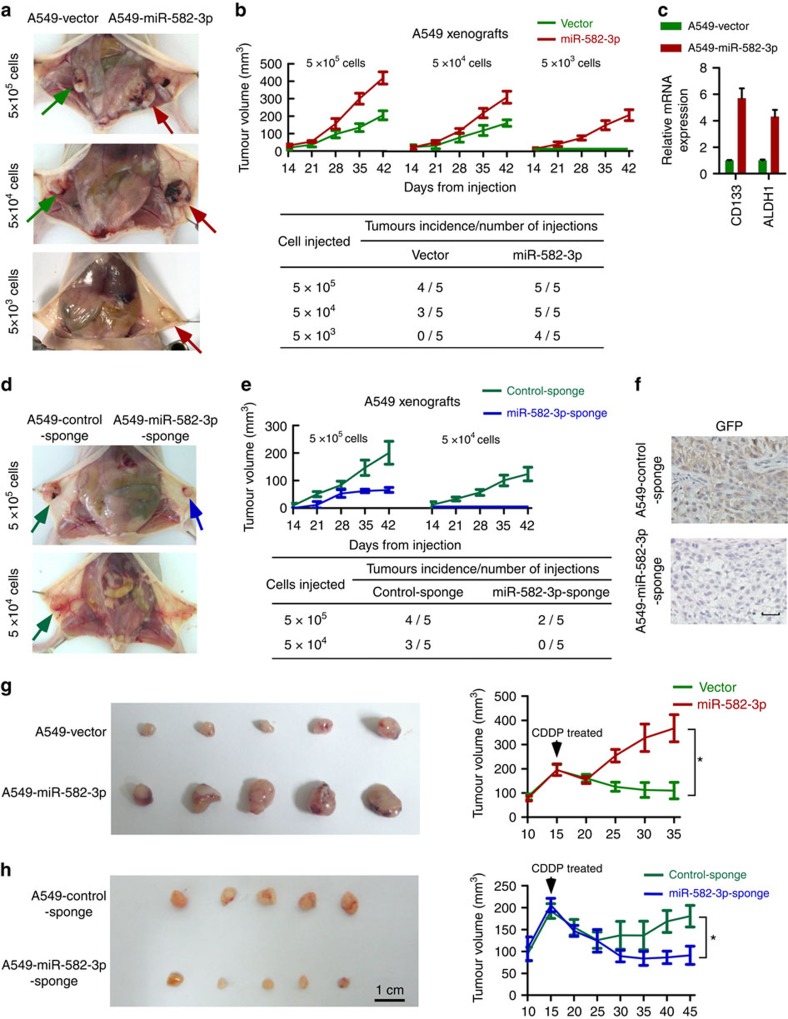
miR-582-3p enhances the tumorigenicity and recurrence of NSCLC *in vivo*. (**a**) In total, 5 × 10^5^, 5 × 10^4^ and 5 × 10^3^ A549-vector (green arrow)- and A549-miR-582 (red arrow)-transfected cells mixed with Matrigel were implanted in BALB/c nude mice (*n*=5 per group). Representative images of the tumours are shown. (**b**) Curves of tumour growth after different numbers of the indicated cells were implanted. Mean tumour volumes are plotted (upper panel). Tumour formation frequencies for different numbers of the indicated cells are shown (lower panel). (**c**) RT–qPCR detection of the expression of CD133 and ALDH1 in the indicated tumour tissues. (**d**) Tumor xenografts inoculated with the indicated numbers of miR-582-sponge- and control-sponge-transduced cells are shown. (**e**) Tumour growth curves (upper panel) and tumour formation frequency (lower panel) in mice inoculated with different numbers of the indicated cells are shown. (**f**) Immunohistochemical staining of GFP protein in paraffin sections of the indicated xenografts confirms the efficacy of silencing miR-582-3p after employment of the miR-582-3p sponge cassette. Scale bar, 50 μm. (**g**) The indicated cells (5 × 10^6^) were injected subcutaneously in nude mice. When the mean tumour volume reached 200 mm^3^, the mice were intravenously injected with 5 mg kg^−1^ CDDP every 2 days for a week. The tumour volume was measured on the indicated days. Scale bar, 1 cm. (**h**) At the experimental endpoint, the tumours were dissected and imaged as indicated. For **b**,**c**,**e** and **h**, the data are presented as the mean±s.e.m. A two-tailed Student's *t*-test was used for statistical analysis (*P<0.05).

To address whether miR-582-3p might play a key role in NSCLC pathogenesis, we established NSCLC cell lines that stably overexpress miR-582-3p and employed a miRNA sponge strategy to silence endogenous miR-582-3p in these cell lines (namely, A549, NCI-H1975 and NCI-H460 cells). As shown in Fig. 2e,f, the absolute miR-582-3p levels were markedly increased in miR-582-3p-overexpressing NSCLC cells compared with the corresponding vector control cells. Marked decreases in GFP intensities in the miR-582-3p-sponge plasmid-transduced cells indicate potent silencing of miR-582-3p (Supplementary Fig. 2). In addition, miR-582-3p-sponge transduction weakly decreased the absolute miR-582-3p levels in H1975, A549 and H460 cells, consistent with previous reports showing that miRNA sponge-mediated silencing causes minimal miRNA degradation^29^. Notably, the absolute miR-582-3p levels in miR-582-overexpressing or miR-582-knockdown NSCLC cells and corresponding vector control cells were within the range of those in human NSCLC tissue samples and were far higher than the physiological miR-582-3p levels in normal lung tissues (Fig. 2e,f). To evaluate the effect of miR-582-3p on the tumorigenicity of NSCLC cells, three doses (5 × 10^5^, 5 × 10^4^ and 5 × 10^3^) of miR-582-3p-overexpressing cells, miR-582-3p-silenced A549 cells, and their corresponding control cells were subcutaneously inoculated in BALB/c nude mice. As shown in Fig. 3a,b, miR-582-3p-transduced cells displayed higher tumorigenicity and increased tumour growth rates than the vector control cells. Notably, only miR-582-3p-overexpressing cells formed visible tumours when 5 × 103 cells were implanted, indicating that miR-582-3p might expand the CSC population in NSCLC cells. Consistently, tumours formed by miR-582-3p-transduced cells showed obviously upregulated levels of cancer stemness-related markers such as CD133 and ALDH1 (Fig. 3c). By contrast, miR-582-3p-silenced cells showed weakened tumorigenicity and slower growth, forming smaller tumours than control cells (Fig. 3d,e). The efficacy of miR-582-3p silencing was confirmed by markedly decreased GFP expression in tumours derived from miR-582-3p-sponge-transduced cells in comparison with those derived from control-sponge-transduced cells (Fig. 3f). Resultant xenografts of miR-582-3p-overexpressing or miR-582-3p-silenced A549 cells and their corresponding control cells inoculated at various densities were histologically confirmed to be lung adenocarcinoma (Supplementary Fig. 3a).

To determine whether the level of miR-582-3p is associated with NSCLC relapse, when the mean tumour volume reached ∼200 mm^3^, mice inoculated with A549-vector and A549-miR-582-3p or A549-control-sponge and A549-miR-582-3p-sponge cells began receiving cisplatin (*cis*-diaminedichloroplatinum, CDDP) treatment. As shown in Fig. 3g, after a transient reduction in mass volume, tumours expressing a higher level of miR-582-3p continued to grow, whereas tumours xenografted with vector control cells failed to regrow in a 35-day period. However, by extending the experimental endpoint to 45 days, tumours formed by control-sponge-transduced cells regrew gradually and slowly, whereas tumours formed by miR-582-3p-silenced cells showed continuous repression. Consequently, xenografts with miR-582-3p-overexpressing cells displayed larger recurrent tumours than those formed by the corresponding control cells. Silencing miR-582-3p led to the opposite effect (Fig. 3h), supporting the notion that miR-582-3p could strongly enhance the tumorigenesis of NSCLC cells and essentially contribute to NSCLC recurrence *in vivo*.

### miR-582-3p promotes a stem cell-like phenotype in NSCLC

**Figure 4:**
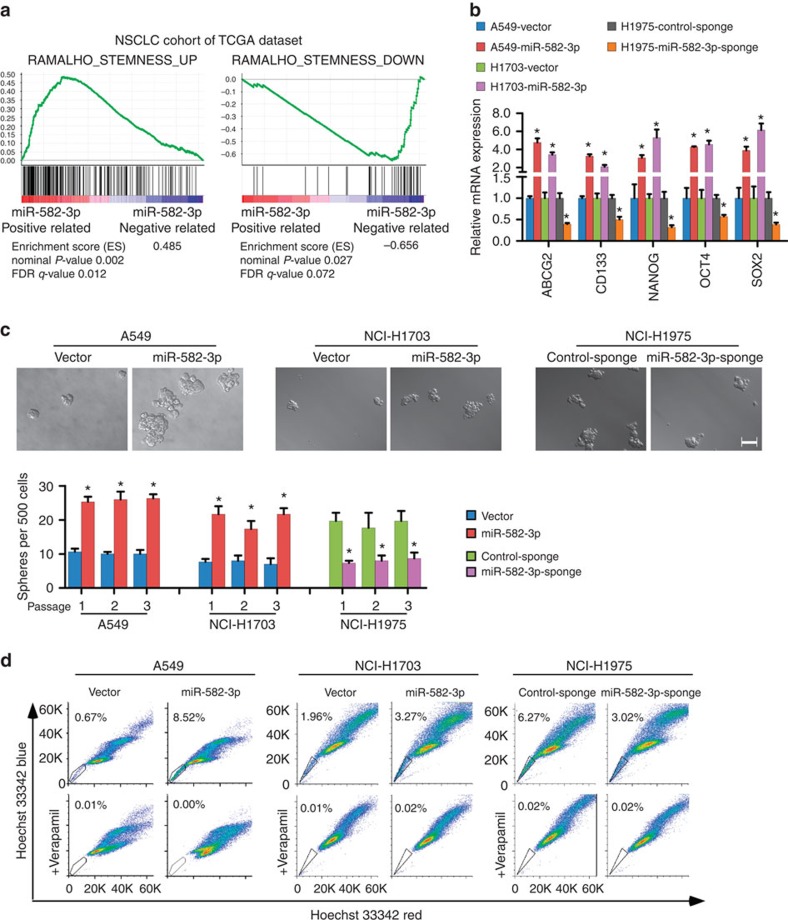
miR-582-3p promotes stem cell-like properties in NSCLC cells *in vitro*. (**a**) GSEA plot showing miR-582-3p expression in association with stemness-related genes. The miR-582-3p level was positively correlated with activated stemness-related gene signatures and inversely correlated with suppressed stemness-related gene signatures in the TCGA lung cancer data set. (**b**) RT–qPCR analysis of the expression levels of cancer stemness-associated markers, including *ABCG2*, *CD133*, *NANOG*, *OCT4*, and *SOX2*, in miR-582-overexpressing and miR-582-silenced cells compared with the corresponding control cells. (**c**) Representative images and quantification of spheres formed by the indicated cells. Scale bar, 100 μm. (**d**) Hoechst 33342 dye exclusion assay showing that overexpressing miR-582-3p increased the SP+ cell proportions in the indicated cells, whereas silencing miR-582-3p decreased these proportions. Each bar represents the mean±s.e.m. derived from three independent experiments. A two-tailed Student's *t*-test was used for statistical analysis (*P<0.05).

We next examined the effects of miR-582-3p overexpression and inhibition on cell proliferation and CDDP-induced cell death *in vitro*. As shown in Supplementary Fig. 3b, neither miR-582-3p overexpression nor inhibition altered the proliferation of NSCLC cells as determined by MTT assay. By contrast, miR-582-3p overexpression promoted the resistance of NSCLC cells to CDDP treatment, and silencing miR-582-3p had the opposite effect, suggesting that miR-582-3p might promote an increase in the CSC population in NSCLC (Supplementary Fig. 3c). As expected, gene set enrichment analysis (GSEA) revealed that miR-582 expression closely correlated with the expression of an *a priori* defined set of stemness-regulating genes^30^ in the TCGA lung cancer data set (Fig. 4a). Notably, the mRNA expression levels of somatic and embryonic stem cell markers related to cancer stemness, including CD133, ABCG2, Nanog, Oct4 and Sox2, were significantly increased in A549 and H1703 cells stably transduced with miR-582-3p but reduced in response to the presence of miR-582-3p-sponge in H1975 cells (Fig. 4b). Furthermore, miR-582-3p-transduced A549, H1703 and H460 cells formed larger and more numerous spheres than vector control cells, whereas miR-582-3p-silenced A549, H1975 and H460 cells formed smaller and fewer spheres compared with control cells (Fig. 4c; Supplementary Fig. 4a). In addition, the proportion of side population (SP) cells was increased in miR-582-3p-overexpressing cells and decreased in miR-582-3p-silenced cells (Fig. 4d; Supplementary Fig. 4b). Taken together, our data suggest that miR-582-3p promotes the stem-like characteristics of NSCLC cells.

### miR-582-3p activates Wnt signalling to promote CSC traits

**Figure 5:**
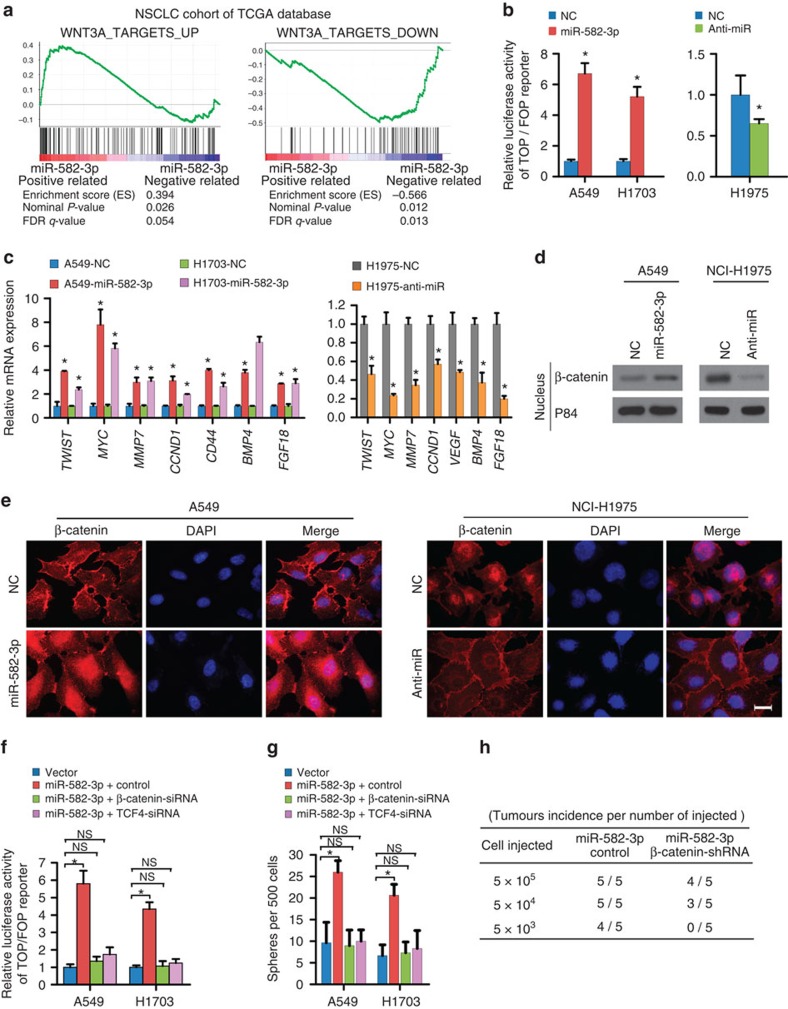
miR-582-3p activates Wnt/β-catenin signalling. (**a**) GSEA plot showing that miR-582-3p expression was positively correlated with Wnt-activated gene signatures and inversely correlated with Wnt-suppressed gene signatures in the TCGA lung cancer data set. (**b**) The indicated cells were transfected with TOP or FOP reporter and *Renilla* pRL-TK plasmids and subjected to dual-luciferase assays 48 h after transfection. The detected reporter activity was normalized to the *Renilla* activity. (**c**) RT–qPCR analysis of the expression of the established downstream targets for the Wnt/β-catenin pathway, including *TWIST*, *MYC*, *MMP7*, *CCND1*, *CD44*, *BMP4* and *FGF18*, in the indicated cells. (**d**) Altered nuclear translocation of β-catenin in response to ectopic miR-582-3p expression. Nuclear fractions of the indicated cells were analysed by western blot analysis. P84 was used as a loading control. (**e**) Subcellular β-catenin localization in the indicated cells was assessed by immunofluorescence staining. Scale bar, 20 μm. (**f**) Luciferase assay of TCF/LEF transcriptional activity in indicated cells. (**g**) Representative images and quantification of cellular spheres formed by the indicated cells. (**h**) Tumour formation frequencies for the different numbers of indicated cells. Each bar represents the mean±s.e.m. derived from three independent experiments. A two-tailed Student's *t*-test was used for statistical analysis (*P<0.05, NS: not statistically significant).

Because the Wnt signalling pathway is considered crucial for the maintenance of cellular stemness and involved in the carcinogenesis of NSCLC, we next examined whether miR-582-3p has an effect on Wnt signalling. By performing GSEA in the TCGA lung cancer data set, we found that the miR-582-3p level was positively correlated with Wnt-activated gene signatures and inversely correlated with Wnt-suppressed gene signatures^31^ (Fig. 5a), suggesting that miR-582-3p might be involved in Wnt/β-catenin signalling activation. Of note, no previously reported functionally meaningful mutations were found in important Wnt signalling components in A549, H1703, H460 or H1975 cell lines (the Catalogue Of Somatic Mutations In Cancer, http://cancer.sanger.ac.uk). Subsequently, we transfected A549 and NCI-H1703 cells with miR-582-3p mimic and inhibitor, respectively, to further examine the effects of miR-582-3p on the Wnt/β-catenin pathway. As shown in Fig. 5b,c, ectopic expression of miR-582-3p in A549 and H1703 cells significantly increased the activity of the luciferase reporter driven by Wnt/β-catenin signals and the expression of seven well-established downstream target genes of the Wnt/β-catenin pathway, whereas the transactivating activity of β-catenin was markedly decreased in response to miR-582-3p silencing in H1975 cells. Meanwhile, subcellular fractionation and immunofluorescence staining assays showed that overexpression of miR-582-3p in NSCLC cells resulted in significant nuclear accumulation of β-catenin, and the opposite results were observed in miR-582-3p-silenced cells (Fig. 5d,e).

Next, we further examined the role of Wnt activation in miR-582-induced stemness. As shown in Fig. 5f–h and Supplementary Fig. 5, silencing β-catenin or TCF4 directly inhibited Wnt/β-catenin signalling and strikingly reversed the ability of miR-582-3p-overexpressing NSCLC cells to form spheres *in vitro* and to initiate tumours *in vivo*, suggesting that miR-582-3p overexpression cannot promote the stemness and tumorigenesis of NSCLC cells without activating Wnt/β-catenin signalling.

### miR-582-3p simultaneously targets AXIN2, DKK3 and SFRP1

**Figure 6:**
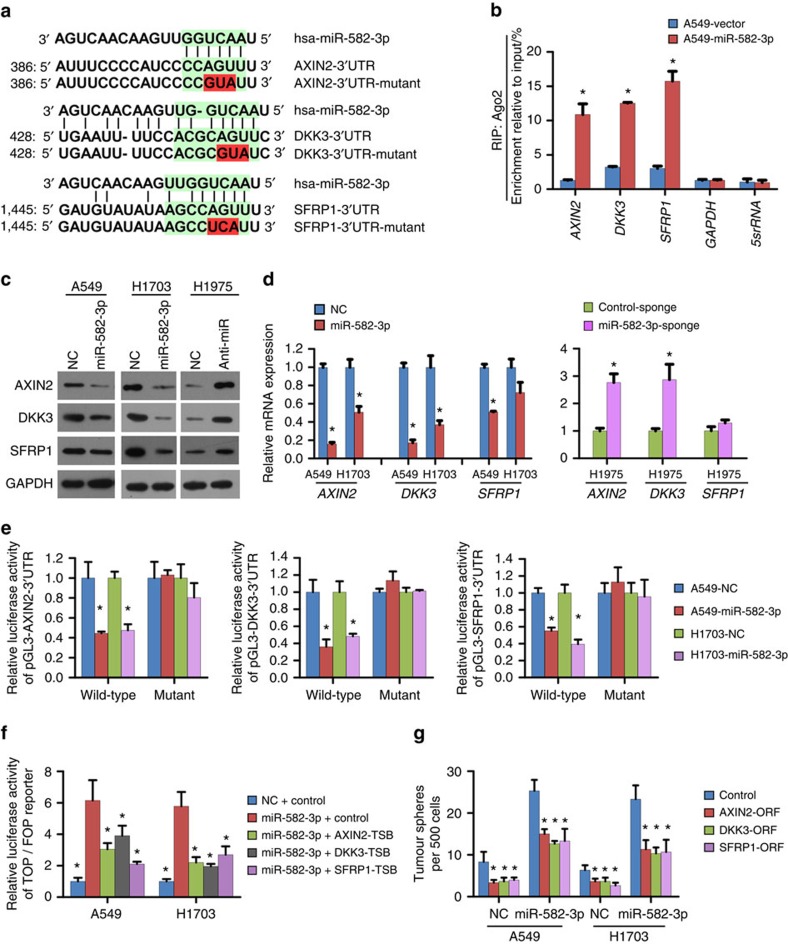
miR-582-3p directly targets multiple negative regulators of the Wnt pathway. (**a**) Predicted binding sites of miR-582-3p in the wild type 3′-UTRs of AXIN2, DKK3 and SFRP1. Mutations in these 3′-UTRs are highlighted in red. (**b**) RIP analysis revealed that AXIN2, DKK3 and SFRP1 mRNAs were recruited to miRNP complexes following immunoprecipitation with Ago2. IgG immunoprecipitation was used as a NC. (**c**,**d**) Western blot and RT–qPCR analyses of AXIN2, DKK3 and SFRP1 expression in the indicated cells. (**e**) Luciferase activity of reporters with wild type or mutant 3′-UTRss of AXIN2, DKK3 and SFRP1 in the indicated cells co-transfected with the indicated oligonucleotides. (**f**) Assay of TOP/FOP luciferase activity in the indicated cells transduced with AXIN2-TSB, DKK3-TSB, SFRP1-TSB or TSB-neg. (**g**) The effect of transducing the ORFs (without 3′-UTRs) of AXIN2, DKK3 or SFRP1 on cellular sphere formation in the indicated control miRNA (NC)- or miR-582-transfected cells. Each bar represents the mean±s.e.m. derived from three independent experiments. A two-tailed Student's *t*-test was used for statistical analysis (*P<0.05).

To investigate the mechanism underlying the robust effect of miR-582-3p on Wnt signalling activation in NSCLC cells, we first sought to identify potential miR-582-3p target genes by employing TargetScan v6.2 and miRanda for miRNA target screening. We identified three tumour suppressor genes associated with Wnt signalling, namely, AXIN2, DKK3 and SFRP1, that could potentially be suppressed by miR-582-3p (Fig. 6a). RNA-immunoprecipitation (RIP) analysis using anti-Ago2 antibody following the overexpression or silencing of miR-582-3p further demonstrated that the mRNAs of these target genes could be specifically recruited to the miRNP complex (Fig. 6b; Supplementary Fig. 6a). Furthermore, western blot and RT–qPCR analyses confirmed that the protein and mRNA levels, respectively, of AXIN2 and DKK3 were drastically reduced in cells engineered to overexpress miR-582-3p but elevated in miR-582-3p-silenced cells (Fig. 6c,d; Supplementary Fig. 6b). Of note, miR-582-3p decreased the SFRP1 protein level but had minimal impact on the SFRP1 mRNA level in H1703 and H1975 cells. By contrast, miR-582-3p decreased both the protein and mRNA levels of SFRP1 in A549 and H460 cells, suggesting that miR-582-3p suppression of target gene expression might involve different mechanisms in different cellular contexts. Subsequently, the 3′-UTRs of these three genes were separately cloned into a luciferase reporter plasmid to detect the direct inhibitory binding of miR-582-3p to the 3′-UTRs. As shown in Fig. 6e, reductions in luciferase activity upon miR-582-3p transfection were observed in A549 and H1703 cells, whereas miR-582-3p overexpression had no suppressive effects on AXIN2, DKK3 or SFRP1 when key mutations were introduced into predicted miR-582-3p target sites in their 3′-UTRs, strongly suggesting that miR-582-3p directly interacts with these three putative targets (Fig. 6a,e). Moreover, we separately transfected three target site blockers (TSBs), namely, AXIN2-TSB, DKK3-TSB and SFRP1-TSB, to specifically inhibit miR-582-3p binding to each of these mRNA targets in vector control and miR-582-overexpressing NSCLC cells. AXIN2-TSB, DKK3-TSB and SFRP1-TSB not only abolished the suppressive effects of miR-582-3p on the 3' UTRs of AXIN2, DKK3 and SFRP1 (Supplementary Fig. 6c), respectively, but also partially reversed the promoting effect of miR-582-3p on Wnt/β-catenin signalling (Fig. 6f), strongly supporting the notion that miR-582-3p does specifically inhibit AXIN2, DKK3 and SFRP1 together and overactivate Wnt/β-catenin signalling.

Furthermore, to reveal the functional significance of miRNA-mediated suppression of AXIN2, DKK3 and SFRP1 in the induction of cellular stemness, we restored AXIN2, DKK3 or SFRP1 expression by transfecting both negative control (NC) miRNA- and miR-582-3p-transfected NSCLC cells with individual open reading frame (ORF) expression cassettes (without their 3′-UTR). As illustrated in Fig. 6g, AXIN2, DKK3 or SFRP1 overexpression inhibited the sphere-forming abilities of both the NC and miR-582-3p-overexpressing NSCLC cells. Furthermore, despite markedly abrogating the sphere-forming abilities of miR-582-3p-overexpressing NSCLC cells, separately restoring AXIN2, DKK3 or SFRP1 expression could not completely reverse these effects, as shown by comparison with the NC cells. This result suggests the significant importance of simultaneous suppression of AXIN2, DKK3 and SFRP1 by miR-582-3p.

### Clinical relevance of miR-582-3p and its targets in NSCLC

**Figure 7:**
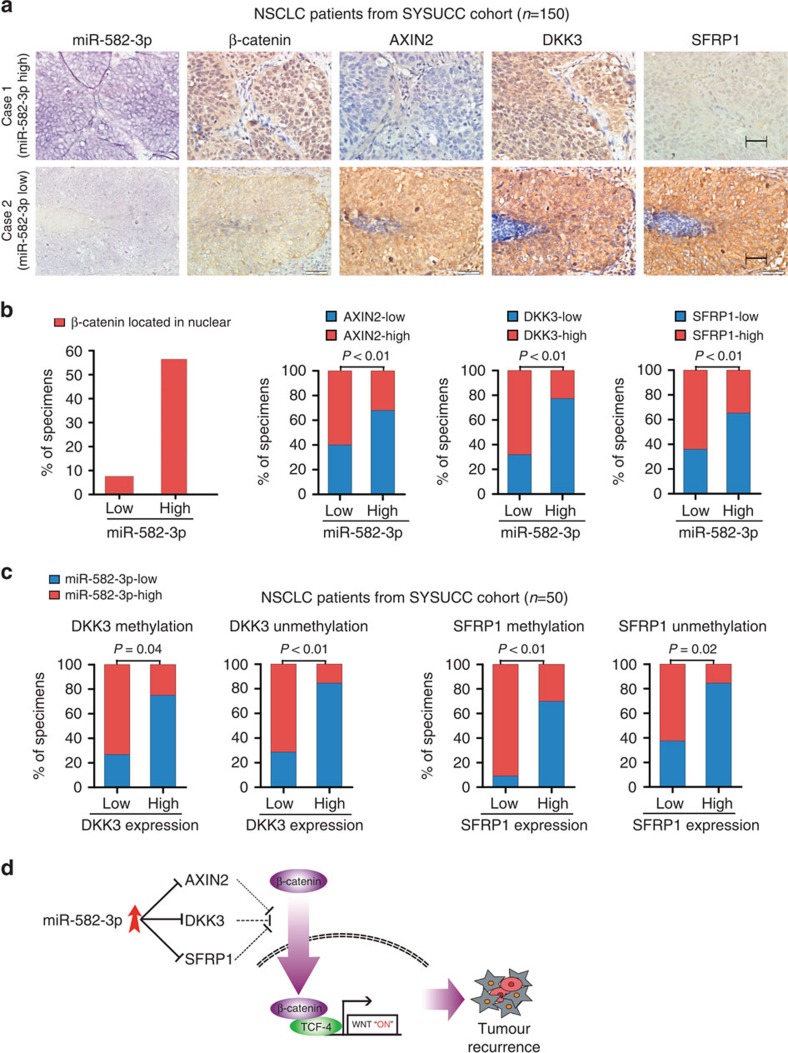
Clinical relevance of miR-582-3p expression with AXIN2, DKK3 and SFRP1 expression in human NSCLC tissue. (**a**) ISH of miR-582-3p and immunohistochemical analysis of β-catenin, AXIN2, DKK3 and SFRP1 expression in serial sections of NSCLC tumour specimens. Scale bar, 50 μm. (**b**) Percentage of specimens showing low or high miR-582-3p expression in relation to the expression levels of AXIN2, DKK3, SFRP1 and nuclear β-catenin. The *χ*^2^-test was used to analyse statistical significance. (**c**) Percentage of specimens showing low or high miR-582-3p expression in relation to DKK3 or SFRP1 expression in 50 NSCLC specimens with unmethylated or methylated DKK3 and SFRP1 promoters. The Fisher's exact test was used to analyse statistical significance. (**d**) Illustration of Wnt activation by miR-582-3p through suppression of multiple Wnt inhibitors.

Last, we examined whether miR-582-3p overexpression and its mediation of Wnt activation were clinically relevant in NSCLC. As shown in Fig. 7a,b, correlation studies in the aforementioned 150 NSCLC clinical specimens of the SYSUCC cohort, which were divided into high (>median) and low (<median) miR-582-3p subgroups each including 75 patients, showed that a higher proportion of tumour specimens expressing high levels of miR-582-3p showed a high level of nuclear β-catenin (based on immunostaining) compared with those with lower miR-582-3p expression (57 vs 8%). An inverse correlation between the expression levels of miR-582-3p and of its targets was also observed in our cohort. Specifically, 68% (51 cases), 77% (58 cases) and 65% (49 cases) of cases with high miR-582-3p expression (75 cases) showed low expression of AXIN2, DKK3 and SFRP1, respectively, whereas 60% (45 cases), 68% (51 cases) and 64% (48 cases) of specimens with low miR-582-3p expression (75 cases) exhibited high levels of AXIN2, DKK3 and SFRP1, respectively (*χ*^2^-test, each *P*<0.05). ISH data, which revealed that the staining pattern of miR-582-3p was typically cytoplasmic, confirmed high and low levels of miR-582-3p, respectively, in the high- and low-miR-582-3p subgroups (Fig. 7a). miR-582-3p quantification by RT–qPCR and ISH staining showed consistent results (Supplementary Table 5), further strengthening the conclusion that miR-582-3p is positively correlated with nuclear β-catenin staining and negatively correlated with AXIN2, DKK3 and SFRP1 expression.

Because DKKs and SFRPs have been documented to be downregulated by promoter hypermethylation in NSCLC, we used methylation-specific PCR (MSP) analysis to examine the methylation status in 50 fresh-frozen NSCLC tissue specimens and found that 23 cases and 21 cases displayed methylation in DKK3 and SFRP1 promoters, respectively, and that 27 cases and 29 cases exhibited unmethylated DKK3 and SFRP1 promoters, respectively. Immunostaining of both DKK3 and SFRP1 and quantification of miR-582-3p in these specimens were performed, and correlations of miR-582-3p with DKK3 and SFRP1 were analysed in specimens with methylated and unmethylated DKK3 and SFRP1 promoters. As shown in Fig. 7c, among 27 cases without hypermethylation in the DKK3 promoter, 10 of 14 cases displaying low DKK3 levels had high miR-582-3p expression and 11 of 13 cases with high DKK3 levels had low miR-582-3p expression. Similarly, for SFRP1, among 29 cases without promoter hypermethylation, 10 of 16 cases showing low SFRP1 levels exhibited high miR-582-3p expression and 11 of 13 cases with high SFRP1 levels exhibited low miR-582-3p expression. In parallel, we also found that miR-582-3p expression was closely inversely correlated with DKK3 and SFRP1 protein levels in 9 cases of NSCLC tissues with unmethylated DKK3 and SFRP1 promoters (Supplementary Fig. 7a–c) Furthermore, significant inverse correlations of miR-582-3p with DKK3 and SFRP1 were observed in specimens with methylated DKK3 and SFRP1 promoters (Fig. 7c). Altogether, these data support the notion that aberrant expression of miR-582-3p is likely to be another important contributor to downregulation of DKK3 and SFRP1, in addition to methylation—silencing of DKK3/SFRP1, in human NSCLC tissues. Overall, our results demonstrate that miR-582-3p activates Wnt signalling by targeting AXIN2, DKK3 and SFRP1; enhances stem cell-like traits; and leads to tumour recurrence and poor prognosis in NSCLC patients (Fig. 7d).

## Discussion

Constitutive activation of Wnt signalling is closely associated with tumour progression in various human cancer types, including NSCLC. Notably, unlike β-catenin and APC mutations in colon cancer, these mutations are very rare in NSCLC^20^,^21^; thus, understanding the biological basis for the observed deregulation of Wnt overactivation is of great value for future development of novel therapeutic strategies. We demonstrate that miR-582-3p overexpression substantially activates and sustains Wnt signalling in NSCLC by simultaneously suppressing multiple Wnt inhibitors in the signalling network, providing a new layer of molecular mechanism by which the Wnt-mediated stem cell-like properties of NSCLC cells are developed.

AXIN2, DKK3 and SFRP1 have been identified as negative regulators of Wnt signalling and suggested to be tumour suppressors in a broad range of human malignancies including NSCLC^14^. AXIN2, a canonical Wnt suppressor, is an important member of the ‘destruction complex' in the cytoplasm. Changes in AXIN2 expression have been linked to poor survival of patients with early-stage lung cancer^15^ DKK3, another Wnt suppressor that we found was targeted by miR-582-3p, is considered a tumour suppressor, and it induces apoptosis in NSCLC. Recently, Lee *et al.* found that DKK3 secreted from activated mesenchymal stem cells (MSCs) could suppress the cell cycle of breast cancer cells, indicating that DKK3 has a paracrine function in the Wnt pathway^32^. SFRP1, which was previously found to directly bind Wnt, suppresses Wnt signalling and has an important role in tumorigenesis^33^,^34^. Overall, these findings indicate that these regulatory molecules contribute to controlling the canonical Wnt pathway extracellularly and cytoplasmically and act in either an autocrine or a paracrine manner. Interestingly, while downregulation of AXIN2, DKK3 and SFRP1 has been extensively observed in lung cancer partly due to DNA hypermethylation^15^,^35^, methylation does not adequately explain their downregulation in NSCLC. For example, DKK3 mRNA was found to be downregulated in over 60% of NSCLC patients^36^,^37^, whereas the proportion of patients with a hypermethylated DKK3 promoter was found to be l<20% (refs 17, 38, 39). Thus, our demonstration that simultaneous suppression of these genes is biologically and clinically attributable to miR-582-3p upregulation reveals a new regulatory mechanism that contributes to the concomitant inactivation of AXIN2, DKK3 and SFRP1 in NSCLC. Due to tight correlation of high miR-582-3p with low DKK3 and SFRP1 expression in NSCLC specimens harbouring either unmethylated or methylated DKK3 and SFRP1 promoters, aberrant expression of miR-582-3p may serve as a novel mechanism underlying the activation of Wnt/β-catenin signalling that is equally as important as methylation in this cancer type.

In the current study, we provide compelling biological and clinical evidence that miR-582-3p expression is markedly upregulated in NSCLC and that miR-582-3p acts to maintain stem cell-like traits and to promote tumorigenesis, chemoresistance and relapse of NSCLC cells, suggesting that miR-582-3p plays an oncogenic role in NSCLC. However, miR-582-3p was previously show to be decreased in clinical samples from patients with high-grade bladder cancer and to negatively regulate tumour progression^40^. Taking these findings and ours together, miR-582-3p appears to play a dual role as both a tumour-promoting and tumour-suppressing miRNA. Similar findings were also noted for other miRNAs, such as miR-98, miR-186, and miR-375, underscoring the need to define the specific role of a certain miRNA in different types of cancer. In such a context, both genetic/epigenetic and microenvironmental cues are believed to contribute to determining whether and how a miRNA molecule functions to promote or to suppress cancer development and progression. Thus, thoroughly investigating the molecular mechanisms mediating the differential biological effects and targets of miR-582-3p in NSCLC and other cancer types remains important. In addition, data regarding DNA copy number variations (CNVs), which are available from the UCSC Cancer Genome Workbench ( https://cgwb.nci.nih.gov/), indicate that the MIR582 gene locus is frequently amplified in 34 of 80 lung cancer cell lines in the GSK data set and in 21 samples from the NCI-60 human tumor cell line panel, suggesting that miR-582-3p overexpression may also be associated with genetic alterations in lung cancer. However, the precise mechanisms underlying the upregulation of miR-582-3p in NSCLC require further investigation.

CSCs play important roles in tumorigenesis, chemoresistance and tumour recurrence, and the ineffectiveness of conventional chemotherapy to eradicate the CSCs frequently result in therapy failure^41^. Therefore, targeting CSCs is a promising therapeutic strategy to address tumor recurrence following resistance to conventional chemotherapy^42^,^43^. The importance of Wnt signalling in the development of stem cell-like properties, which is well defined in a wide variety of cancer types, suggests that targeting aberrant Wnt signalling activity in CSCs may represent an important approach to cancer therapy^44^,^45^. In fact, gene therapies delivering DKK3 and AXIN2, which were demonstrated to be targets of miR-582-3p in this study, are being clinically tested in prostate cancer and colon cancer, respectively (NCT01197209, NCT01882660). In addition, analogues of the CBP/β-catenin antagonist ICG-001, which is able to target and eliminate drug-resistant CSCs in several types of tumour models including leukaemia, glioblastoma and colon cancer, are currently being tested in a phase I clinical trial^44^. However, effective small-molecule drugs targeted against Wnt signalling usually act against only a single molecule^44^,^46^. Mounting evidence suggests that miRNAs are promising molecules for use in therapeutic interventions likely because miRNAs function as master regulators of cellular genes and exert strong regulatory effects on tumor development and progression by targeting multiple molecules. The first miRNA anti-cancer drug, MRX34, a ‘mimic' of the tumour suppressor miR-34, has already advanced into a phase I clinical trial in patients with unresectable liver cancer (NCT01829971). Our current study revealed that forced expression of miR-582-3p in lung cancer cells might subject a subset of tumour cells to the pool of CSCs by causing simultaneous inhibition of AXIN2, DKK3 and SFRP1 and constitutive activation of the Wnt signalling pathway, thus promoting tumorigenesis and relapse of NSCLC xenografts. Meanwhile, antagonizing miR-582-3p caused multilevel inactivation of Wnt signalling and had obvious inhibitory effects on CSCs and tumorigenesis. These results suggest that miR-582-3p may be a novel prototype therapeutic agent that can target Wnt signalling in lung CSCs to suppress tumorigenesis and relapse.

Interestingly, previous studies also identified other miRNAs that were indicative of NSCLC patient prognosis. Notably, in our current study, miR-582-3p exhibited positive correlations with both poor prognosis and early recurrence in patients with NSCLC. In addition, miR-582-3p showed potential for application as a useful prognostic indicator, as evidenced in two cohorts of patients with NSCLC. Whether and how miR-582-3p and other miRNAs identified thus far are associated with patient outcome and whether they can be used separately or together in the clinic needs to be further investigated with large-scale, prospective clinical trials.

## Methods

### Cell culture

Primary NLECs were obtained in accordance with the rules and regulations concerning ethical issues regarding the experimental use of human subjects in China. Prior patient consent and approval from the Institutional Research Ethics Committee of SYSU were obtained. NLECs were primarily cultured according to a previous report^47^. In brief, surgically resected specimens of normal lung tissue were promptly removed and transported aseptically in Hanks' solution (Invitrogen, Carlsbad, CA) supplemented with 100 units per ml penicillin, 100 μg ml^−1^ streptomycin (Invitrogen) and 5 μg ml^−1^ gentamycin (Invitrogen). The tissue specimens were then incubated with 1.5 units per ml dispase (Roche Molecular Biochemicals, Indianapolis, IN) at 4 °C overnight, and the epithelium was dissected and incubated with trypsin (Invitrogen). The reaction was stopped with soybean trypsin inhibitor (Sigma, Saint Louis, MI) and centrifuged, followed by resuspension in keratinocyte-SFM medium (KSFM) supplemented with 40 μg ml^−1^ bovine pituitary extract, 1.0 ng ml^−1^ EGF, 100 units per ml penicillin, 100 μg ml^−1^ streptomycin, 5 μg ml^−1^ gentamycin and 100 units per ml nystatin (Invitrogen). The BEAS-2B immortalized human bronchial epithelial cell line (Shanghai Institutes of Biological Sciences, Shanghai, China) was cultured in LHC-9 medium as instructed by the provider. Lung cancer cell lines, including A549, 95D, SK-MES-1, NCI-H460, NCI-H358, NCI-H1650, NCI-H1299, NCI-H1703 and NCI-H1975, were obtained from the cell banks of the Shanghai Institutes of Biological Sciences (Shanghai, China) or from Fu Erbo Biotechnology Co., Ltd. (Guangzhou, China). The cell lines were maintained in DMEM (Invitrogen) supplemented with 10% fetal bovine serum (HyClone, Logan, UT) and 1% penicillin/streptomycin (Invitrogen). All cell lines were authenticated by short tandem repeat fingerprinting at IDEXX RADIL and the SYSU Forensic Medicine Lab.

### TCGA data analysis

Level 3 miRNA-Seq and RNA-Seq data from 539 lung tumour samples with available OS data were downloaded from the TCGA lung cancer data set portal ( https://tcga-data.nci.nih.gov/; accessed on 2013/7/3; TCGA accession codes are listed in Supplementary Data 1). Among these 539 lung tumour samples, 139 samples also had RFS data. Clinical information regarding the enrolled patient specimens is presented in Supplementary Table 1. Analyses were performed using BRB-ArrayTools, developed by Dr Richard Simon and the BRB-ArrayTools Development Team^48^. A given miRNA is excluded under any of the following conditions: <20% of the expression data show at least a 1.5-fold change in either direction from the miRNA's median value or the percentage of data missing or filtered out exceeds 50%. After filtering out, 354 miRNAs remained for analysis.

### Tissue specimens

The current study was conducted on 150 paraffin-embedded, archived NSCLC tumour specimens, which were histopathologically and clinically diagnosed at the SYSUCC from 2001 to 2006. These specimens are defined as the SYSUCC cohort. Clinical information regarding the research subjects is summarized in Supplementary Table 2. NSCLC tissue and paired adjacent non-cancerous lung tissue specimens were stored frozen in liquid nitrogen until further use. Adjacent non-tumour tissues were obtained from a standard distance (3 cm) from resected neoplastic tissues of patients with NSCLC who underwent surgical lung resection and confirmed by pathological evaluation. Prior patient consent and approval from the Institutional Research Ethics Committee of SYSU were obtained for the use of these clinical materials for research purposes.

### Significance analysis of microarray survival analysis

Microarray data were assessed using MeV4.9 ( http://www.tm4.org/mev/) with the SAM program (Stanford University, Stanford, CA) and censored survival data^49^. SAM identifies the genes that are most closely correlated with survival time and uses permutation analysis to estimate the FDR.

### Gene set enrichment analysis

Global mRNA expression profiles of a subset of TCGA lung cancer specimens for which miR-582-3p expression data were available were subject to GSEA to identify the association of miR-582-3p with stemness related and WNT3A-mediated Wnt/β-catenin signalling pathways, using methods described in previous reports^30^,^31^ For GSEA, miR-582-3p expression was treated as a numeric variable. We applied a continuous-type cls file of the miR-582-3p profile to phenotype labels in GSEA. The metric for ranking genes in GSEA was set as ‘Pearson', and the other parameters were set to their default values^50^. GSEA was performed using GSEA 2.0.9 software ( http://www.broadinstitute.org/gsea/).

### Plasmids and transfection

A DNA fragment containing the hsa-miR-582 precursor with 500 bp of flanking genomic sequence on each side was inserted into the retroviral transfer plasmid pMSCV-puro. The ORFs of AXIN2, DKK3 and SFRP1 were cloned into the mammalian expression vector pcDNA 3.1 (Invitrogen). The 3′-UTRs of AXIN2, DKK3 and SFRP1 were amplified and cloned downstream of the luciferase gene in a modified pGL3 control vector (Promega). miR-582-3p mimics, anti-miR-582 oligonucleotides and their corresponding control oligonucleotides were purchased from RiboBio (Guangzhou, China). Custom-designed TSBs for blocking miR-582-3p binding to AXIN2, DKK3 and SFRP1, namely, AXIN2-TSB, DKK3-TSB and SFRP1-TSB, and non-targeting control TSB (TSB-neg) LNA oligonucleotides were purchased from Exiqon (Vedbaek, Denmark). Transfection of plasmids or oligonucleotides was performed using Lipofectamine 2000 reagent (Invitrogen) according to the manufacturer's instructions.

### miRNA sponge

miRNA sponges are transcripts with repeated antisense miRNA sequences that can sequester endogenous miRNAs as targets^29^. In this study, synthetic oligonucleotides containing six tandem repeats of ‘bulged' miR-582-3p-binding sites were ligated into the 3′-UTR of GFP mRNA in a lentiviral plasmid, named ‘GFP-miR-582-3p-sponge' to determine the effects of miRNA modulation. The control plasmid, ‘GFP-control-sponge', contained a sequence with no homology to any human gene sequences. Sequence of miR-582-3p-sponge: 5′-ACTAGTTAAGGTTCAGTTGCGACACCAGTTACGATGGTTCAGTTGCGACACCAGTTACGATGGTTCAGTTGCGACACCAGTTACGATGGTTCAGTTGCGACACCAGTTACGATGGTTCAGTTGCGACACCAGTTACGATGGTTCAGTTGCGACACCAGTTAGGATCC-3′. Sequence of control-sponge: 5′-ACTAGTTAATGTTAGCTAAGAGAAAACTTCGATTGTTAGCTAAGAGAAAACTTCGATTGTTAGCTAAGAGAAAACTTCGATTGTTAGCTAAGAGAAAACTT CGATTGTTAGCTAAGAGAAAACTTCGATTGTTAGCTAAGAGAAAACTTCGC GGATCC-3′.

### Western blot analysis

Western blot analysis was performed according to a previously described standard method^51^ using anti-AXIN2 (1:500; Abcam, Cambridge, MA), anti-DKK3 (1:500; GeneTex, San Antonio, TX), anti-SFRP1 (1:500; Abcam) or anti-β-catenin (1:1,000; Cell Signaling, Danvers, MA) antibodies. Blotted membranes were stripped and re-blotted with anti-GAPDH rabbit monoclonal antibody (1:2000; Sigma, St. Louis, MO) as a loading control. Original data of immunoblotting are presented in Supplementary Fig. 8.

### RNA extraction and RT–qPCR

Total miRNA was isolated from cultured cells and surgically resected fresh NSCLC tissues using a mirVana miRNA Isolation Kit (Ambion, Austin, TX). Total miRNA was extracted from paraffin-embedded, archived clinical NSCLC specimens using a RecoverAll Total Nucleic Acid Isolation kit (Ambion) according to the manufacturer's instructions. The miRNA levels were assayed with Taqman probes and primer sets (Applied Biosystems, Foster City, CA, USA) in accordance with the manufacturer's instructions. To perform absolute quantification in the miRNA analyses, an HPLC-purified synthetic oligoribonucleotide standard that was identical in sequence to hsa-miR-582-3p was purchased (Invitrogen), and the exact concentration of this standard was measured at A260. On the basis of the measured concentration, samples containing absolute copy numbers were prepared by serial dilution. These serially diluted standards were reverse transcribed and assayed by RT–qPCR to generate a standard curve in parallel with the samples. The cycle threshold (Ct) values of each sample for miR-582-3p were converted into the corresponding copy number based on standard-curve analysis. Extraction of total RNA and measurement of mRNA quantity were performed as described previously^52^. RNA was extracted from cells using TRIzol (Invitrogen) following protocols supplied by the manufacturer. First-strand cDNA was generated by MMLV transcriptase (Promega) using random primers. Real-time RT–PCR was performed on a CFX96 real-time PCR detection system (Bio-Rad), and a Roche SYBR FAST Universal qPCR Kit (Roche Molecular Biochemicals) was used for gene detection. The sequences of primers are listed in Supplementary Table 6.

### Northern blot analysis

Northern blot analysis was performed according to standard methods as previously described^53^. In brief, total RNA (20 μg) isolated from human NSCLC cell lines and paraffin-embedded NSCLC specimens was dissolved in 2 × RNA loading buffer (95% formamide, 0.025% SDS, 0.025% bromophenol blue, 0.025% xylene cyanol, 0.025% ethidium bromide and 0.5 mM EDTA), heated at 95 °C for 3 min, loaded onto denaturing 15% TBE-urea gels and transferred onto positively charged nylon membranes (Roche, Penzberg, Germany). Northern blots were prehybridized at 65 °C for 1 h using hybridization buffer (Roche) and subjected to hybridization with 3′-DIG-labelled RNA probe (100 ng ml^−1^) targeted against miR-582-3p (Exiqon) overnight at room temperature. Probe detection was performed using a DIG Luminescent Detection Kit (Roche, Penzberg, Germany) according to the manufacturer's protocol. After the blots were equilibrated in detection buffer, they were incubated with the chemiluminescent substrate CDP-Star and used to expose Kodak Biomax MR film.

### Sphere formation assay

In total, 500 cells were seeded in 6-well ultra-low cluster plates (Corning Inc., Corning, NY) for 10 days. Spheres were cultured in DMEM/F12 serum-free medium (Invitrogen) supplemented with 2% B27 (BD Pharmingen, Carlsbad, CA), 20 ng ml^−1^ EGF, 20 ng ml^−1^ bFGF, 0.4% BSA, and 5 μg ml^−1^ insulin (Sigma).

### Flow cytometric analysis

Cells were dissociated with trypsin, resuspended at 1 × 10^6^ cells per ml in DMEM containing 2% FBS and subsequently preincubated at 37 °C for 30 min with or without 100 μM verapamil (Sigma) to inhibit ABC transporters. Next, the cells were incubated for 90 min at 37 °C with 5 μg ml^−1^ Hoechst 33342 (Sigma). Finally, the cells were incubated on ice for 10 min and washed with ice-cold PBS before flow cytometric analysis. Flow cytometry data were analysed by FlowJo 7.6.1 software.

### Luciferase reporter assay

Cells were seeded in triplicate in 24-well plates and allowed to settle for 24 h. The indicated plasmids plus 1 ng of pRL-TK *Renilla* plasmid were transfected into the cells using Lipofectamine 2000 reagent (Invitrogen). Forty-eight hours after transfection, dual-luciferase reporter assays by determining the enzyme activities of luciferase using a BioTek Synergy2 microplate reader (Bio-Tek, Winooski, VT) at wavelengths of 560 and 465 nm following the manufacturer's instructions (Promega) as previously described^54^.

### RNA immunoprecipitation

Co-immunoprecipitation (Co-IP) of miRNP with anti-Ago2 (Abcam) or IgG (Sigma) was performed as previously described^55^. Cells were co-transfected with HA-Ago2 together with 100 nM miR-582-3p, and lysed in buffer containing 100 mM KCl, 5 mM MgCl_2_, 10 mM HEPES (pH 7.4) and 0.5% NP-40, followed by HA-Ago2 immunoprecipitation using HA-antibody; and the immune complex captured by protein A agarose was washed six times in buffer containing 150 mM KCl, 5 mM MgCl_2_, 10 mM HEPES (pH 7.4) and 0.1% NP-40 for six times. RNA extraction was performed using an RNAeasy Kit (Qiagen, Valencia, CA). The IP material was analysed by RT–qPCR to test the association of AXIN2, DKK3 or SFRP1 mRNA with miR-582-3p in the RNA-induced silencing complex (RISC). GAPDH and 5S rRNA were both used as NCs.

### Methylation-specific PCR

MSP was performed according to a previously described standard method. Genomic DNA was extracted from tissue using a QIAamp DNA Mini Kit (Qiagen, Hilden, Germany). After spectrophotometric quantitation, 1 μg of genomic DNA was bisulfite-treated using an EpiTect Bisulfite Kit (Qiagen, Hilden, Germany) according to the manufacturer's instructions. For the MSP, primer pairs were designed to target the methylated or unmethylated promoters of DKK3 and SFRP1 as previously referenced^38^,^56^ and listed in Supplementary Table 7.

DNA from normal lymphocytes (NLs) was used as the NC, *in vitro* methylated DNA (IVD) was used as the positive control (PC), and reagents mixed with pure water instead of DNA was used as the reagent control (RC). All MSP products were analysed by electrophoresis on 2% agarose gels.

### Tumour xenografts *in vivo*

All experimental procedures were approved by the Institutional Animal Care and Use Committee of SYSU. BALB/c nude mice (6–7 weeks of age, 18–20 g, female) were randomly divided into 3 groups (*n*=5 per group). The indicated cells were mixed with Matrigel (BD Pharmingen, Carlsbad, CA) (final concentration, 25%) and inoculated into the inguinal folds of the mice at three doses (5 × 10^5^, 5 × 10^4^, 5 × 10^3^). The mice were anesthetized and killed 42 days after inoculation, and the tumours were removed, weighed and sectioned. In the tumour relapse analysis, the mice were inoculated with 5 × 10^6^ cells for 15 days and treated with CDDP (5 mg kg^−1^) every 2 days for a week. At the experimental endpoint, the animals were euthanized, and the tumours were excised and subjected to pathological examination. Tumour volume was calculated using the equation (L × W^2^)/2.

### *In situ* hybridization (ISH)

ISH was performed using an LNA miRNA ISH optimization kit (Exiqon, Vedbaek, DK) for formalin-fixed, paraffin-embedded kidney samples according to the protocol provided by the manufacturer. Briefly, 10-μm thick sections from frozen lung tumours that were mounted on slides were fixed in 4% paraformaldehyde. Then, the slides were incubated with 15 mg ml^−1^ proteinase K (Exiqon) for 20 min at 37 °C. After the slides were dehydrated, they were hybridized with a double digoxygenin (DIG)-labelled LNA miR-582-3p probe (80 nM) for 1 h at 55 °C. The slides were washed with SSC buffer and then incubated with blocking solution for 15 min, followed by incubation with anti-DIG reagent for 60 min at room temperature. After the slides were washed, the probe signal was visualized with anti-DIG antibodies and NBT/BCIP solution.

### Immunohistochemistry

Immunohistochemistry assays were performed on the paraffin-embedded NSCLC tissue^57^, using the following primary antibodies: anti-AXIN2 (1:100; Abcam), anti-DKK3 (1:100; GeneTex), anti-SFRP1 (1:100; Abcam) and anti-β-catenin (1:200; Cell Signaling). The degree of immunostaining of indicated proteins was evaluated and scored by two independent observers, who scored both the proportions of tumour cells that stained positively and the intensity of the staining. The proportion of positively stained tumour cells was graded as follows: 0 (no positive tumour cells); 1 (<10% positive tumour cells); 2 (10–50% positive tumour cells); and 3 (>50% positive tumour cells). The intensity of the staining was graded as follows: 0 (no staining); 1 (weak staining=light yellow); 2 (moderate staining=yellow brown); and 3 (strong staining=brown). The staining index (SI) was calculated as the product of the staining intensity × the percentage of positive tumour cells, resulting in scores of 0, 1, 2, 3, 4, 6 and 9. Cutoff values for high and low expression of the protein of interest were chosen based on a heterogeneity measurement using the log-rank test with respect to OS. An SI score ≥4 was considered high expression, and an SI score ≤3 was considered low expression.

### Statistical analysis

All statistical analyses, except for the analyses of microarray data, were performed using the SPSS 20.0 statistical software package. Sample size was determined by power analysis to achieve a minimum effect size of 0.5 with a *P* value of <0.05. The patients were divided into two groups according to whether they exhibited a high (>the median) or low (≤the median) miR-582-3p expression level. Survival curves were analysed by the Kaplan–Meier method, and a log-rank test was used to assess significance. Univariate and multivariate survival analyses were performed using Cox regression analysis. The *χ*^2^-test or Fisher's exact test was used to analyse the relationship between the expression levels of miR-582-3p and target genes. Comparisons between groups were performed using Student's *t*-test. All error bars represent the mean±s.e.m. derived from three independent experiments. Data analysis was performed by two independent investigators who were blinded to the sample groups. *P* values <0.05 were considered statistically significant.

## Additional information

**How to cite this article:** Fang, L. *et al.* Aberrantly expressed miR-582-3p maintains lung cancer stem cell-like traits by activating Wnt/β-catenin signalling. *Nat. Commun.* 6:8640 doi: 10.1038/ncomms9640 (2015).

## Supplementary Material

Supplementary Information Supplementary Figures 1-8 and Supplementary Tables 1-7

Supplementary Data 1 Includes accession codes for 539 lung tumor samples from TCGA lung cancer dataset

## Figures and Tables

**Table 1:** Significance analysis of microarrays (SAM) using the TCGA lung cancer data set identified microRNAs associated with patients' OS or RFS.

**microRNAs**	***q*****-value (%)**
*High expression correlated with shorter RFS*	
hsa-miR-582	0.00
hsa-miR-196a-2	0.00
	
*Low expression correlated with shorter RFS*	
hsa-miR-1468	0.00
hsa-let-7c	8.02
hsa-miR-99a	8.02
hsa-miR-187	8.02
hsa-miR-195	8.02
hsa-miR-221	8.02
hsa-miR-432	8.02
hsa-miR-654	8.02
	
*High expression correlated with shorter OS*	
hsa-miR-582	0.00
hsa-miR-450a-2	0.00
hsa-miR-503	0.00
	
*Low expression correlated with shorter OS*	
hsa-miR-29c	0.00
hsa-miR-181c	0.00
hsa-miR-181d	0.00

RFS, Recurrence-free survival; OS, overall survival.
