# Supplementary material for: Aberrantly expressed miR-582-3p maintains lung cancer stem cell-like traits by activating Wnt/β-catenin signalling
Source: Nat Commun. 2015 Oct 15;6:8640. doi: 10.1038/ncomms9640 (PMC4667703; doi:10.1038/ncomms9640)
Supplement: Supplementary Information — Supplementary Figures 1-8 and Supplementary Tables 1-7 [file ncomms9640-s1.pdf]

Supplementary Information

Supplementary Figure 1

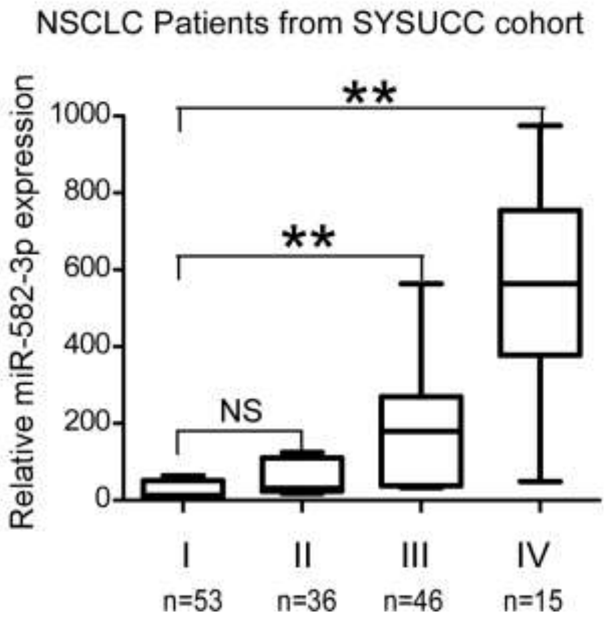

Supplementary Figure 1. Expression of miR-582-3p in NSCLC tumor tissues correlates with clinical staging. Expression levels of miR-582-3p in the indicated NSCLC patients at each clinical stage (I-IV) as determined by RT-qPCR. Transcript quantities were normalized to U6 expression. For the Box-Whisker diagrams, the lower and upper bounds of the boxes represent the lower and upper quartiles, respectively; lines within the boxes represent the median values; and whiskers denote the minimum and maximum values in the indicated data sets. Each bar represents the mean±s.e.m. derived from three independent experiments. A two-tailed Student's *t*-test was used for statistical analysis (**: P<0.01, NS: not statistically significant).

Supplementary Figure 2

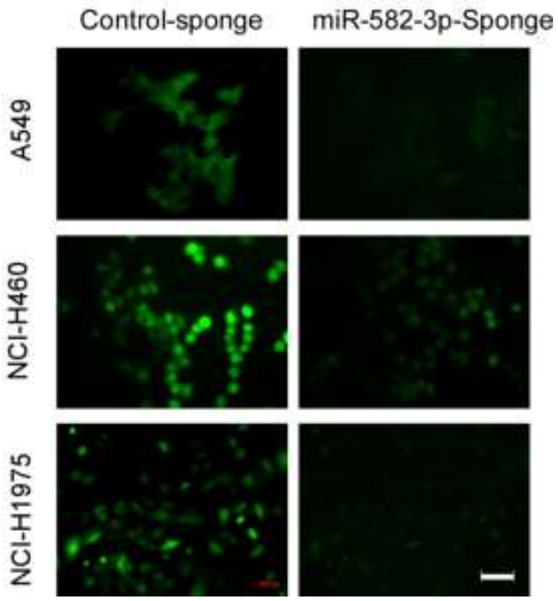

Supplementary Figure 2. The effect of miR-582-3p silencing by miR-582-sponge.

Fluorescent visualization of A549, NCI-H460 and NCI-H1975 cells transduced with GFP-control-sponge or pSin-GFP-miR-582-sponge plasmid confirms efficient inhibition of miR-582-3p. Scale bar: 100 μm.

Supplementary Figure 3

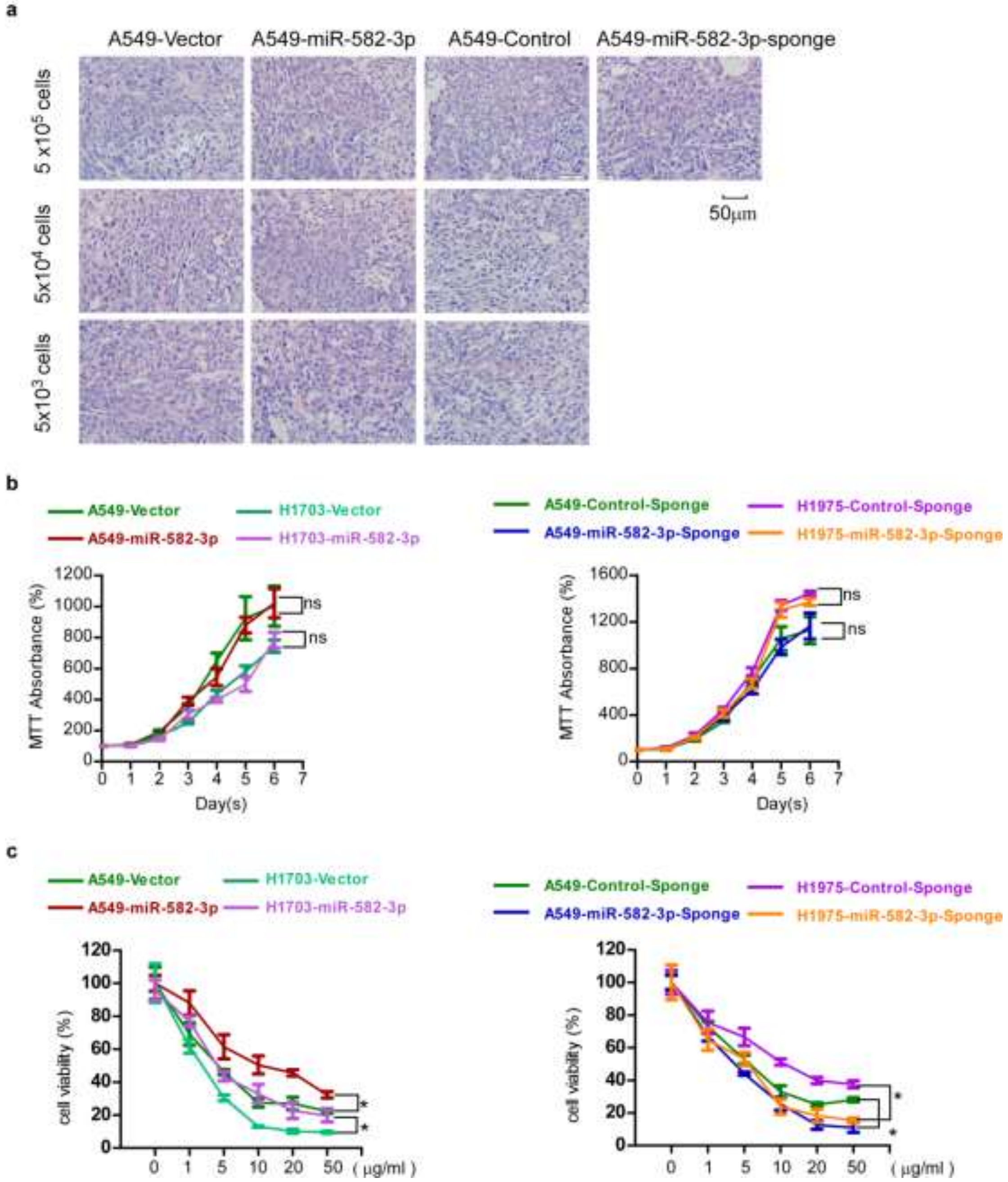

Supplementary Figure 3. miR-582-3p promotes resistance of NSCLC cells to CDDP treatment. (a) Histological confirmation of xenografted NSCLC tumors by hematoxylin/eosin (H&E) staining in the indicated groups. Scale bar: 50 μm. **(b)** Growth curves of the indicated cells as determined by MTT assay. **(c)** MTT assays revealed the number of viable cells of each indicated cell line treated with the indicated concentration of CDDP. Each bar represents the mean±s.e.m. derived from three independent experiments. A two-tailed Student's *t*-test was used for statistical analysis (*: *P*<0.05, ns: not statistically significant).

Supplementary Figure 4

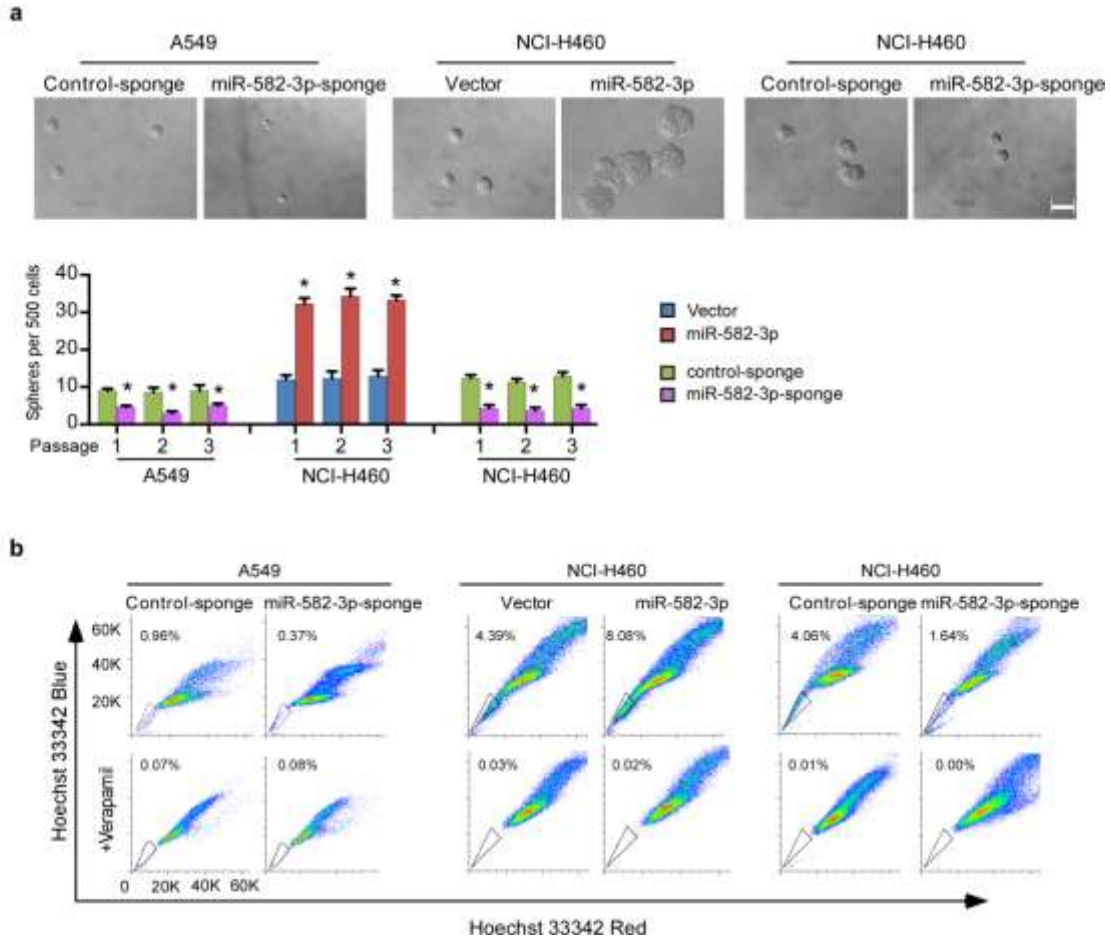

Supplementary Figure 4. miR-582-3p promotes stem cell-like properties in NSCLC cells *in vitro*. **(a)** Representative images and quantification of cellular spheres formed by the indicated cells. Each bar represents the mean±s.e.m. derived from three independent experiments. A two-tailed Student's *t*-test was used for statistical analysis (*: $P<0.05$). Scale bar: 100 μm. **(b)** Hoechst 33342 dye exclusion assay showing the proportions of SP+ cells in the indicated cells.

Supplementary Figure 5

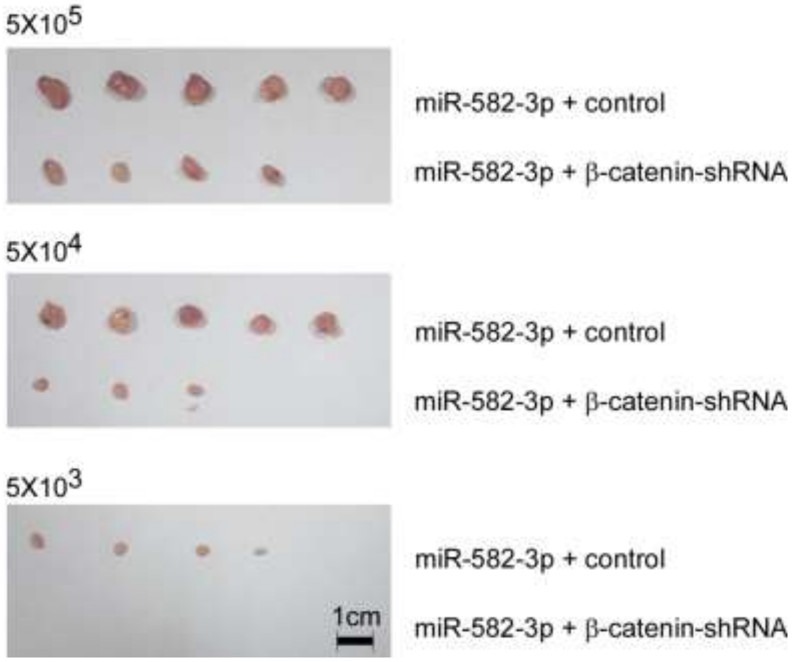

Supplementary Figure 5. Silencing β-catenin suppresses the tumorigenesis of miR-582-3p-overexpressing NSCLC cells. Excised xenografts derived from the indicated cells at various cell densities are shown. Scale bar: 1 cm.

Supplementary Figure 6

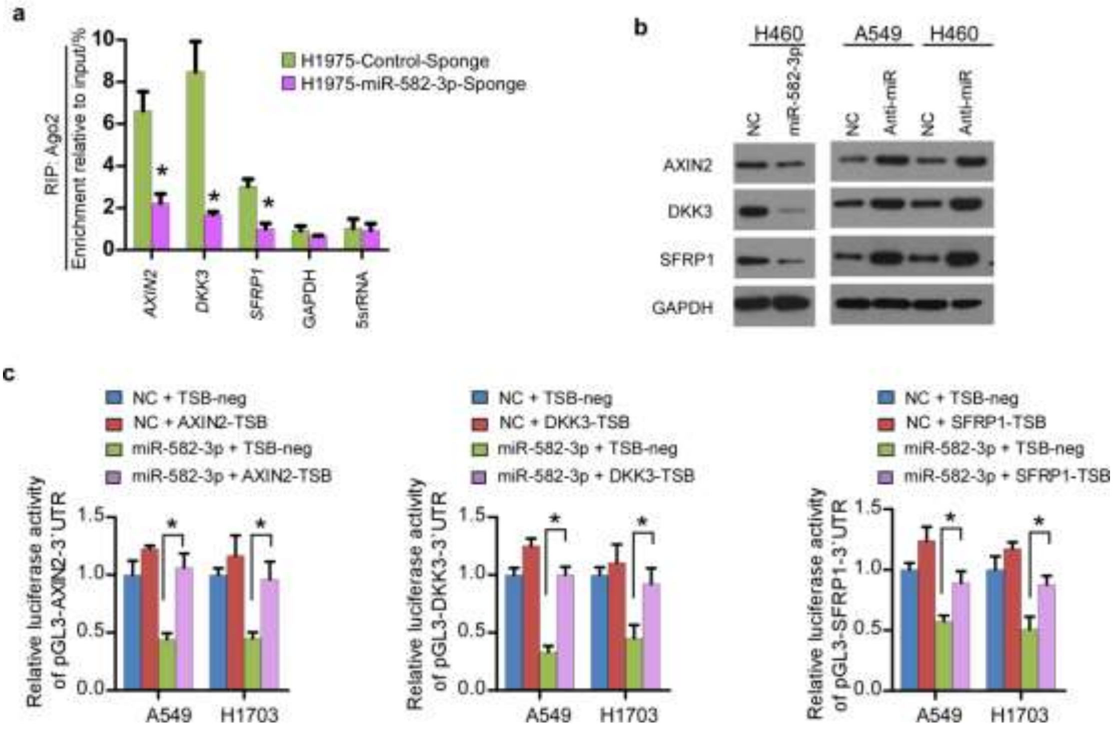

Supplementary Figure 6. miR-582-3p directly targets AXIN2, DKK3 and SFRP1. **(a)** Enrichment of the 3'UTRs of AXIN2, DKK3 and SFRP1 in Ago2-containing miRNP complexes detected by RT-qPCR following immunoprecipitation using an antibody against Ago2. The results are presented as normalized fold enrichment. **(b)** Western blot analysis of AXIN2, DKK3 and SFRP1 expression in the indicated cells. **(c)** Luciferase assay of the 3' UTR activity of AXIN2, DKK3 and SFRP1 in the indicated cells transfected with the indicated TSB. Each bar represents the mean±s.e.m. derived from three independent experiments. A two-tailed Student's *t*-test was used for statistical analysis (*: $P<0.05$).

Supplementary Figure 7

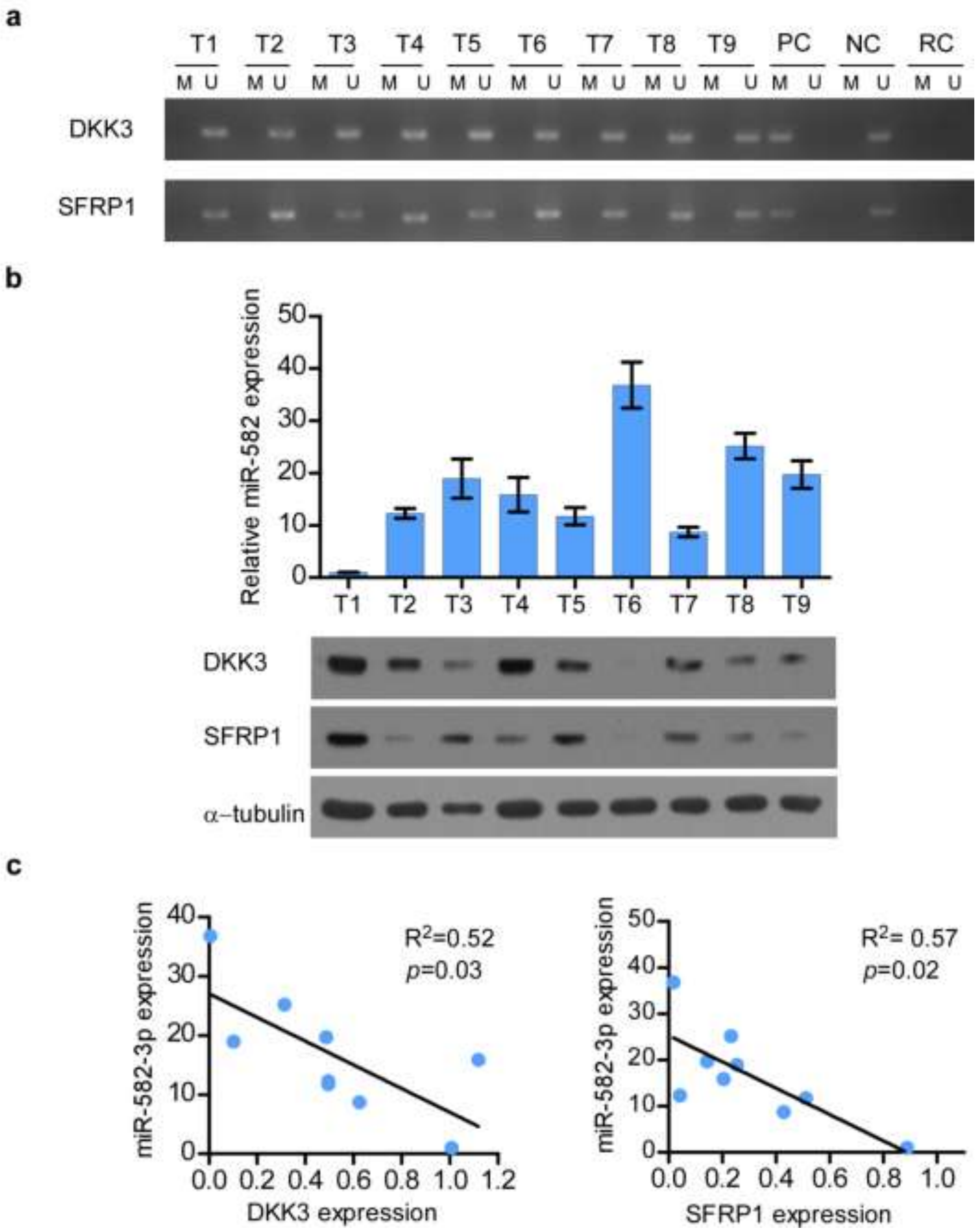

Supplementary Figure 7. miR-582-3p expression negatively correlates with DKK3 and SFRP1 expression in NSCLC specimens in which both DKK3 and SFRP1 promoters are unmethylated. **(a)** Nine specimens with unmethylated DKK3 and SFRP1 promoters were detected by MSP. **(b)** RT-qPCR analysis of miR-582-3p expression and western blot analysis of DKK3 and SFRP1 expression in the 9 specimens. **(c)** Correlation analysis of miR-582-3p expression with DKK3 and SFRP1 levels in the 9 specimens.

Figure 2e

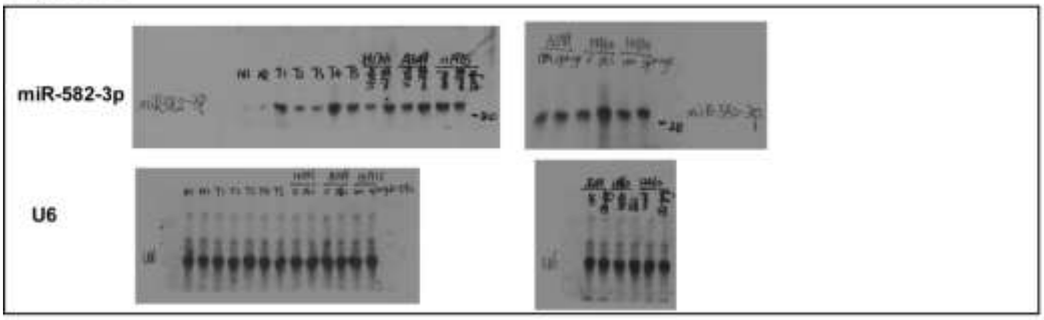

Figure 5d

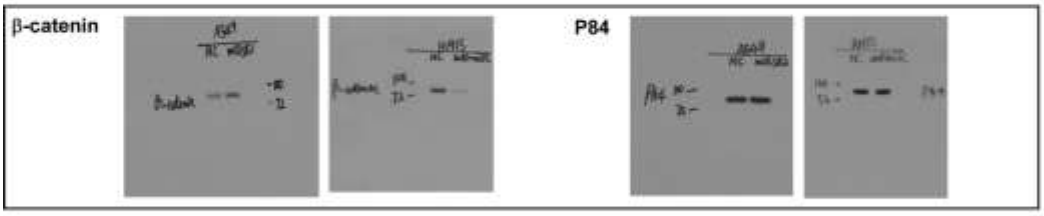

Figure 6c and Supplementary Figure 6b

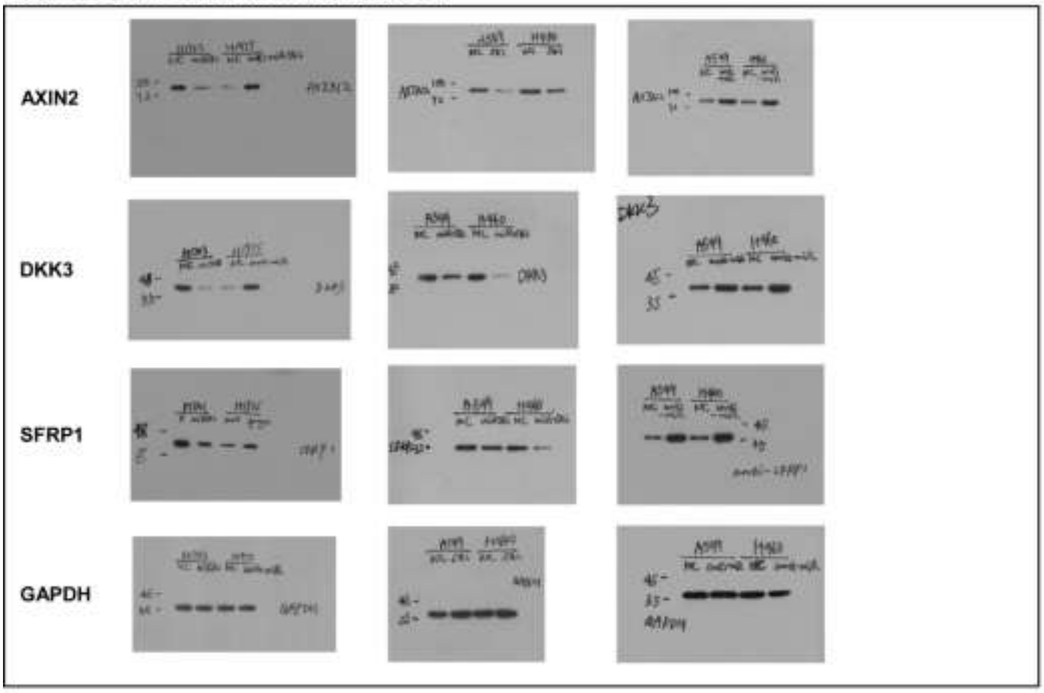

Supplementary Figure 7b

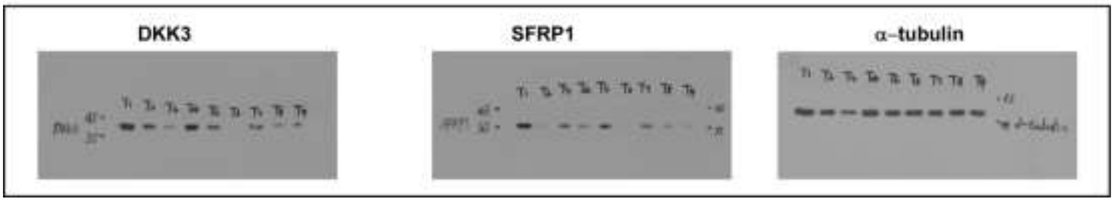

Supplementary Figure 8. Uncropped scans of all western blots presented in Figures and supplementary Figures.

Supplementary Table 1. Clinicopathological Characteristics of Studied Patients and Expression of miR-582 in NSCLC from TCGA Dataset

Terms	No. of cases	Percentage
**Age (years)**		
≥Median	291	54.0%
<Median	248	46.0%
**Gender**		
Male	312	57.9%
Female	227	42.1%
**Pathologic Type**		
LUAD(1)	346	64.2%
LUSC(2)	193	35.8%
**Chemotherapy**		
YES	136	25.2%
NO	403	74.8%
**Tobacco Smoking History**		
Reformed Smoker	327	60.7%
Current Smoker	139	25.8%
Non-smoker	55	10.2%
**miR-582 expression**		
miR-582 High	270	50.1%
miR-582 Low	269	49.9%

Terms	No. of cases	Percentage
**Clinical Stage**		
Stage I	287	53.2%
Stage II	145	26.9%
Stage III	86	16.0%
Stage IV	18	3.30%
**Tumor Size**		
T1	163	30.2%
T2	302	56.0%
T3	55	10.2%
T4	18	3.30%
**Nodes Pathologic**		
N0	350	64.9%
N1	109	20.2%
N2	69	12.8%
N3	1	0.20%
**Metastasis Pathologic**		
M0	364	67.5%
M1	17	3.20%

LUAD(1) : lung adenocarcinoma
LUSC(2) :lung squamous cell carcinoma

Supplementary Table 2. Clinicopathological Characteristics of Studied Patients and Expression of miR-582 in NSCLC from the SYSUCC Cohort

Terms	No. of cases	Percentage	Terms	No. of cases	Percentage
**Age(y)**			**T Classification**		
≥Median	75	50.0%	T1	19	12.7%
<Median	75	50.0%	T2	79	52.7%
**Gender**			T3	44	29.3%
male	117	78.0%	T4	8	5.33%
female	33	22.0%	**N Classification**		
**Pathologic Type**			N0	81	54.0%
LUSC	62	41.3%	N1	34	22.7%
LUAD	73	48.7%	N2	34	22.7%
Adenosquamous Carcinoma	15	10.0%	N3	1	0.67%
**Clinical Staging**			**Distant Metastasis**		
Stage I	53	35.3%	YES	15	10.0%
Stage II	36	24.0%	NO	135	90.0%
Stage III	46	30.7%	**Therapy**		
Stage IV	15	10.0%	NO	104	69.3%
**miR-582 expression**			YES	46	30.7%
miR-582 High	75	50.0%			
miR-582 Low	75	50.0%			

Supplementary Table 3. Univariate and Multivariate Analysis of Various Clinical Parameters in Patients with NSCLC by Cox-Regression Analysis from TCGA Dataset

	Univariate analysis		Multivariate analysis	
	**HR(95%CI)**	***P* value**	**HR(95%CI)**	***P* value**
**Age(y)**				
< Median	1.00		1.00	
≥ Median	1.14 (0.79-1.65)	0.476	1.37(0.91-2.04)	0.128
**Gender**				
male	1.00		1.00	
female	0.91(0.63-1.30)	0.604	0.96 (0.64-1.44)	0.851
**Pathologic Type**				
LUAD(1)	1.00		1.00	
LUSC(2)	0.79 (0.54-1.15)	0.224	0.71 (0.47-1.09)	0.315
**T Classification**				
T1	1.00		1.00	
T2	1.63(1.02-2.60)	0.042	1.53(0.93-2.50)	0.092
T3	1.31(0.56-3.06)	0.539	1.35(0.56-3.22)	0.504
T4	2.54(1.13-5.70)	0.024	1.53(0.61-3.81)	0.362
**N Classification**				
N0	1.00		1.00	
N1	1.24(0.79-1.93)	0.352	1.21 (0.76-1.93)	0.413
N2、N3	2.24 (1.44-3.48)	0.001	2.37(1.46-3.86)	0.001
**Distant Metastasis**				
No	1.00		1.00	
Yes	1.81(0.88-3.74)	0.106	1.87(0.83-4.25)	0.133
**Tobacco Smoking History**				
Non-smoker	1.00		1.00	
Reformed Smoker	1.01(0.49-2.08)	0.986	1.40 (0.63-3.10)	0.407
Current Smoker	0.92(0.41-2.05)	0.838	1.44(0.59-3.49)	0.424
**Chemotherapy**				
NO	1.00		1.00	
YES	1.21(0.80-1.81)	0.366	1.02(0.65-1.59)	0.948
**miR-582 Expression**				
Low	1.00		1.00	
High	1.70(1.17-2.45)	0.005	1.85(1.25-2.73)	0.002

LUAD(1) : lung adenocarcinoma
LUSC(2) :lung squamous cell carcinoma

Supplementary Table 4. Univariate and Multivariate Analysis of Various Clinical Parameters in Patients with NSCLC by Cox-Regression Analysis from SYSUCC Cohort

	Univariate analysis		Multivariate analysis	
	HR(95%CI)	*P* value	HR(95%CI)	*P* value
**Age(y)**				
< Median	1.00		1.00	
≥ Median	1.47(0.96-2.24)	0.073	1.50(0.97-2.33)	0.069
**Gender**				
male	1.00		1.00	
female	0.65(0.38-1.11)	0.114	0.67(0.37-1.19)	0.173
**Pathologic Type**				
LUAD	1.00		1.00	
LUSC	0.90(0.57-1.41)	0.642	0.97(0.59-1.61)	0.919
Adenosquamous carcinoma	1.80(0.93-3.50)	0.082	2.23(1.08-4.60)	0.030
**T Classification**				
T1	1.00		1.00	
T2	3.11(1.23-7.85)	0.016	2.57(0.99-6.70)	0.053
T3	4.57(1.78-11.73)	0.002	3.28(1.20-8.95)	0.021
T4	4.16(1.20-14.41)	0.024	2.20(0.55-8.81)	0.264
**N Classification**				
N0	1.00		1.00	
N1	1.74(1.03-2.95)	0.040	1.61(0.88-2.93)	0.122
N2 N3	2.98(1.81-4.88)	<0.001	1.96(1.12-3.43)	0.018
**Distant Metastasis**				
No	1.00		1.00	
Yes	2.07(1.13-3.82)	0.019	1.63(0.79-3.38)	0.187
**Chemotherapy**				
NO	1.00		1.00	
YES	1.05(0.67-1.65)	0.829	0.75(0.46-1.21)	0.241
**miR-582 Expression**				
Low	1.00		1.00	
High	2.85(1.83-4.44)	<0.001	2.26(1.39-3.68)	0.001

Supplementary Table 5. miR-582-3p expression analysis correlation between RT-qPCR and *in situ* hybridization.

		in situ hybridization		Total
		miR-582-Low (score<6)	miR-582-High (score≥6)	
qRT-PCR	miR-582-Low (<median)	68	7	75
	miR-582-High (>median)	4	71	75
Total		72	78	150

χ^2=106 r = 0.86 P < 0.001

Supplementary Table 6. The sequences of primers used in Real-time RT–PCR assay.

Gene Symbol	Real-time-primer-up	Real-time-primer-down
DKK3	CAGGTGTACTGGAAGCTGGC	TCACATCTGTGGGAGACGAA
AXIN2	CTGGTGCAAAGACATAGCCA	AGTGTGAGGTCCACGGAAAC
SFRP1	GGCTTCTTCTTCTTGGGGAC	ATCTCTGTGCCAGCGAGTTT
TWIST1	TCCATTTTCTCCTTCTCTGGAA	CCTTCTCGGTCTGGAGGAT
MYC	CACCGAGTCGTAGTCGAGGT	TTTCGGGTAGTGGAAAACCA
MMP7	GCATCTCCTTGAGTTTGGCT	GAGCTACAGTGGGAACAGGC
CCND1	GGCGGATTGGAAATGAACTT	TCCTCTCCAAAATGCCAGAG
CD44	CACGTGGAATACACCTGCAA	GACAAGTTTTGGTGGCACG
BMP4	GCATTCGGTTACCAGGAATC	TGAGCCTTTCCAGCAAGTTT
FGF18	CTTACGGCTCACATCGTCC	ACTTCCTGCTGCTGTGCTTC

Supplementary Table 7. The sequences of primer pairs detecting the methylated or unmethylated promoters of DKK3 and SFRP1

Methylated DKK3 primers	5′-GGCGTTAGGGGCGGGCGGC-3′
	5′-GCTCTGCGCCCGCAACCGCCG-3′
Unmethylated DKK3 primers	5′-GGGTGTTAGGGGTGGGTGGTG-3′
	5′-CCACTCTACACCCACAACCACCA-3′
Methylated SFRP1 primers	5′-TGTAGTTTTCGGAGTTAGTGTCGCGC-3′
	5′-CCTACGATCGAAAACGACGCGAACG-3′
Unmethylated SFRP1 primers	5′-GTTTTGTAGTTTTTGGAGTTAGTGTTGTGT-3′
	5′-CTCAACCTACAATCAAAAACAACACAAACA-3′
